# Clinical utility of genetic testing in 201 preschool children with inherited eye disorders

**DOI:** 10.1038/s41436-019-0722-8

**Published:** 2019-12-18

**Authors:** Eva Lenassi, Jill Clayton-Smith, Sofia Douzgou, Simon C. Ramsden, Stuart Ingram, Georgina Hall, Claire L. Hardcastle, Tracy A. Fletcher, Rachel L. Taylor, Jamie M. Ellingford, William D. Newman, Cecilia Fenerty, Vinod Sharma, I. Chris Lloyd, Susmito Biswas, Jane L. Ashworth, Graeme C. Black, Panagiotis I. Sergouniotis

**Affiliations:** 1 Division of Evolution and Genomic Sciences, School of Biological Sciences, Faculty of Biology, Medicines and Health, University of Manchester, Manchester, UK; 2 Manchester Centre for Genomic Medicine, Saint Mary’s Hospital, Manchester University NHS Foundation Trust, Manchester, UK; 3 Manchester Royal Eye Hospital, Manchester University NHS Foundation Trust, Manchester, UK; 4 Great Ormond Street Hospital for Children NHS Foundation Trust, London, UK

**Keywords:** clinical utility, inherited eye disease, congenital cataract, inherited retinal disease, albinism

## Abstract

**Purpose:**

A key property to consider in all genetic tests is clinical utility, the ability of the test to influence patient management and health outcomes. Here we assess the current clinical utility of genetic testing in diverse pediatric inherited eye disorders (IEDs).

**Methods:**

Two hundred one unrelated children (0–5 years old) with IEDs were ascertained through the database of the North West Genomic Laboratory Hub, Manchester, UK. The cohort was collected over a 7-year period (2011–2018) and included 74 children with bilateral cataracts, 8 with bilateral ectopia lentis, 28 with bilateral anterior segment dysgenesis, 32 with albinism, and 59 with inherited retinal disorders. All participants underwent panel-based genetic testing.

**Results:**

The diagnostic yield of genetic testing for the cohort was 64% (ranging from 39% to 91% depending on the condition). The test result led to altered management (including preventing additional investigations or resulting in the introduction of personalized surveillance measures) in 33% of probands (75% for ectopia lentis, 50% for cataracts, 33% for inherited retinal disorders, 7% for anterior segment dysgenesis, 3% for albinism).

**Conclusion:**

Genetic testing helped identify an etiological diagnosis in the majority of preschool children with IEDs. This prevented additional unnecessary testing and provided the opportunity for anticipatory guidance in significant subsets of patients.

## INTRODUCTION

Inherited eye disorders are an important cause of visual impairment in children and young adults.^1^ Prevalent subtypes include inherited retinal disease (IRD), pediatric cataracts, ocular anterior segment dysgenesis (ASD), and albinism. These conditions may manifest as isolated ophthalmic disorders (nonsyndromic forms) or as part of multisystemic syndromes that include extraocular features (syndromic forms). In the latter scenario, ocular manifestations are often one of the first presenting features of a syndrome (e.g., individuals with Marfan syndrome often present with ectopia lentis).^2–6^

In the past three decades, over 400 genes have been found to be associated with inherited eye disorders. Such advances in delineating the molecular pathology of these conditions, together with breakthroughs in DNA sequencing technologies, catalyzed the development of powerful genomic tests.^3,7^ These tests have revolutionized diagnostics for many inherited eye disorders and, in 2012, the American Academy of Ophthalmology published guidelines encouraging their routine use for a number of these conditions.^8^ Despite this, variation in the current provision of genetic testing remains significant.^9^ Considering economic factors is necessary but insufficient and one way to address this variation is by continuing to accrue robust evidence of benefit.

In 2017, the US National Academies of Sciences, Engineering, and Medicine published a report on how evidence on the usefulness of genetic tests is generated and evaluated.^10^ A key concept in this document is clinical utility, defined as “the ability of a test to improve clinical outcomes measurably and add value for patient management decision making compared with current management without genetic testing.”^10^ Over the past decades, this term has been construed both narrowly and broadly. In its narrowest sense, it refers to the ability of a test to lead to an improved health outcome (e.g., impact on mortality, morbidity, or visual disability). A broader definition includes any change in management (e.g., preventing additional investigations or introducing surveillance measures) while the broadest definition encompasses any outcome that is important to the affected individual or family (e.g., early resolution of uncertainty, better understanding of the condition, effect on reproductive or life planning).^9,11,12^ The primary focus of previous studies has been the evaluation of specific test parameters such as analytical validity (i.e., the ability to accurately identify variants of interest; associated with technical performance) and clinical validity (i.e., the ability to detect the clinically defined disorder of interest; associated with the diagnostic yield).^3,7,13–15^ As a result, data on how genetic tests influence patient management and health outcomes in different clinical scenarios are scarce.

The aim of this study was to assess the current clinical utility of diagnostic genetic testing in infants and young children (aged 0–5 years) with different inherited eye disorders (including bilateral pediatric cataracts, bilateral ectopia lentis, bilateral ASD, albinism, and IRD). The potential to inform management and to allow the definition of specific care pathways is highlighted.

## MATERIALS AND METHODS

### Participants

Study subjects were retrospectively ascertained through the database of the North West Genomic Laboratory Hub, Manchester, UK. Only children who were diagnosed through the tertiary pediatric ophthalmic genetics service at Manchester University NHS Foundation Trust, Manchester, UK were included. The families who chose to undergo genetic analysis were offered pretest counseling and provided written informed consent.

Probands selected for study were unrelated and had to meet the following inclusion criteria:

- To be five years of age or less at the time of referral for genetic testing.
- To be referred for genetic testing between September 2011 and August 2018 (i.e., over a 7-year period).
- To have one of the following clinical diagnoses: bilateral pediatric cataracts, bilateral ectopia lentis, bilateral ASD (including pediatric primary glaucoma), albinism (ocular or oculocutaneous), and IRD.

A subset of the cases included in this study has been previously reported (see Supplementary Table 1 for more information).^2,3^ Ethics committee approval for the study was obtained from the North West Research Ethics Committee (11/NW/0421 and 15/YH/0365) and all investigations were conducted in accordance to the tenets of the Declaration of Helsinki.

### Phenotypic data collection

The clinical notes and/or electronic health-care record entries were reviewed for each study participant. The clinical impression at the time of referral for genetic testing was recorded. The outcome of the consultation in which the genetic test results were discussed was also noted (including information on referrals to other subspecialties and discussions around prognosis and family planning).

A full clinical history was obtained for each proband and all study subjects were examined by a consultant ophthalmologist. A subset of participants underwent fundus imaging, and electrodiagnostic testing was performed in most children with a suspected diagnosis of albinism or IRD using previously described methods.^2^ Where extraocular features were present or suspected, a full systemic assessment was undertaken by a consultant clinical geneticist.

### Clinical genetic testing and bioinformatic analysis

Blood samples were obtained from all probands and DNA was extracted. Multigene panel testing and analysis were subsequently performed at the North West Genomic Laboratory Hub (ISO 15189:2012; UKAS Medical reference 9865). DNA samples were processed using Agilent SureSelect (Agilent Technologies, Santa, Clara, CA) target enrichment kits designed to capture all exons and 50 base pairs of flanking intronic sequences of selected panels of genes. The decision on which panel to use was made by the referring clinician (either a consultant pediatric ophthalmologist or a consultant clinical geneticist with an interest in ophthalmic genetics). In general, the following panels were available (see also Supplementary Tables 2–7):

- For pediatric cataract and ectopia lentis: either 114^3^ (*n* = 46) or 144 (*n* = 31) or 12 (*n* = 1) genes.
- For ASD: either 114^3^ (*n* = 3) or 45 (*n* = 22) or 32 (*n* = 2) genes.
- For suspected albinism: 18 (*n* = 30) or 26 (*n* = 1) or 40 (*n* = 1) genes.
- For IRD: either 105^16^ (*n* = 14) or 177^17^ (*n* = 43) genes.

Notably, 7 probands underwent genome sequencing using a previously described approach.^15^ Information on the exact panel test that was performed in each study participant can be found in Supplementary Table 1. Additionally, 18 probands with a diagnosis of syndromic inherited eye disease were tested using a DNA microarray assay while 4 probands had cytogenetic chromosomal analysis. A list of all the relevant transcripts/genes can be found in Supplementary Tables 2–7.

Sequencing, bioinformatic analyses, and clinical interpretation were performed as previously described.^2,3,15–17^ A pathogenicity classification score was assigned to each rare variant after in silico modeling and appraisal of the scientific literature. A clinical report was then generated and variants that could account for the tested individual’s phenotype were highlighted. Reports were then discussed in monthly multidisciplinary team meetings.

For the purpose of this study, subjects were split into two groups:

- Probable molecular diagnosis group: probands with clearly or likely pathogenic variant(s) in an apparently disease-causing state (e.g., ≥1 variant in a gene linked with dominant disease or ≥2 variants in a gene linked with recessive disease).
- Unknown molecular diagnosis group: all other probands.

We defined “diagnostic yield” as the percentage of individuals with a particular phenotype who received a probable molecular diagnosis.^10^

## RESULTS

Overall, 201 (120 male, 81 female) unrelated infants and young children (0–5 years at the time of genetic testing) met the inclusion criteria for the study. Median age at referral for genetic testing was 3 years. The breakdown of the 201 study participants among high order diagnostic categories is shown in Table 1. Extraocular features were noted at the time of referral for genetic testing in 33/201 cases (16%) while a relevant family history was recorded at the time of referral for genetic testing in 31/201 cases (15%).

**Table 1:** Breakdown among diagnostic categories, and percentage of study participants in each category for which a probable molecular diagnosis^a^ could be identified.

Diagnostic category	Number of probands	Diagnostic yield of genetic testing
Bilateral pediatric cataracts	74	50% (37/74)
Bilateral ectopia lentis	8	75% (6/8)
Bilateral ocular anterior segment dysgenesis (including pediatric primary glaucoma)	28	39% (11/28)
Albinism	32	91% (29/32)
Inherited retinal disease	59	78% (46/59)

^a^We considered probands to have probable molecular diagnosis when they carried clearly or likely pathogenic variant(s) in an apparently disease-causing state (e.g., ≥1 variant in a gene linked with dominant disease or ≥2 variants in a gene linked with recessive disease). Further information can be found in “Materials and Methods” and in Supplementary Table 1.

### Diagnostic yield

A probable molecular diagnosis was identified in 129/201 probands (64%); the diagnostic yield of genetic testing for each condition is shown in Table 1 and the genetic findings are presented in detail in Supplementary Table 1. There was significant genetic heterogeneity. Genes found to be mutated in ≥1% of the cohort (i.e., in >2 probands) are shown in Table 2. Defects in these 14 genes were collectively responsible for disease in 71/201 probands (35%). In contrast, 33 genes were associated with disease in a single family each (33/201 probands; 16%).

**Table 2:** Genes found to be mutated in ≥1% of the cohort.

Gene name	Number of probands in the whole cohort	Number of probands in the relevant diagnostic category
*TYR*	18/201	18/32
*OCA2*	7/201	7/32
*CNGB3*	7/201	7/59
*CACNA1F*	6/201	6/59
*COL4A1*	4/201	4/74
*CRYAA*	4/201	4/74
*CEP290*	4/201	4/59
*LTBP2*	3/201	3/8
*CYP1B1*	3/201	3/28
*FOXE3*	3/201	3/28
*NYX*	3/201	3/59
*RDH12*	3/201	3/59
*RS1*	3/201	3/59
*CRYBA1*	3/201	3/74

Among the individuals reported to have extraocular features at the time of referral for genetic testing, a probable molecular diagnosis was identified in 13/33 (39%). For individuals with a known family history, the diagnostic yield was 23/31 (74%).

### Clinical utility

The genetic test result informed/altered management in 66/201 probands (33%). The following scenarios were identified:

- Avoiding additional unnecessary tests.
- Initiating surveillance for extraocular manifestations (referral to another subspecialty).
- Reducing prognostic uncertainty (distinguishing stationary from progressive conditions).
- Determining eligibility for clinical trials of gene-based therapeutic interventions.

The number of individuals impacted per diagnostic category is shown in Table 3. More details on the exact management change for each patient can be found in Supplementary Table 1. Two illustrative cases are presented in the following section.

**Table 3:** Number of preschool children with suspected inherited eye disorders for whom genetic testing lead to a clear management change.

Diagnostic category	Avoiding unnecessary tests	Initiating surveillance for extraocular manifestations	Reducing prognostic uncertainty	Determining eligibility for gene-based therapeutic trials
Bilateral pediatric cataracts	50% (37/74)^a^	12% (9/74)	–	–
Bilateral ectopia lentis	50% (4/8)	25% (2/8)	–	–
Bilateral ocular anterior segment dysgenesis (including pediatric primary glaucoma)	4% (1/28)	4% (1/28)	–	–
Albinism	–	3% (1/32)	–	–
Inherited retinal disease	–	8% (5/59)	25% (15/59)	3% (2/59)
Overall	21% (42/201)	9% (18/201)	7% (15/201)	1% (2/201)

Further information can be found in the illustrative cases presented in “Results” and in Supplementary Table 1.

^a^This figure is based on the fact that there was no need to perform the conventional biochemical tests for individuals with a known molecular diagnosis.

Most probands had no known family history of relevance (170/201; 85%). In these simplex cases the inheritance pattern is unclear without a molecular diagnosis, and genetic testing is required to refine the risk to future pregnancies and family members. Among the simplex cases who were found to have a probable molecular diagnosis (106/170), 69% (73/106) had autosomal recessive, 23% (24/106) had autosomal dominant, and 8% (9/106) had X-linked inheritance (Supplementary Table 8). Six study participants were shown to have a de novo variant in a gene associated with autosomal dominant or X-linked inheritance (which suggested that the risk to future pregnancies of the parents is low).

### Two illustrative cases

Proband 18014933 was diagnosed with bilateral dense nuclear cataracts at day 1 of age. She was otherwise a healthy infant and there was no family history of visual problems. Lensectomy procedures were performed at 7 weeks of age. Genetic testing was requested prior to surgery and the c.2317G>A p.(Gly773Arg) variant was identified in the *COL4A1* gene in heterozygous state. Testing of parental samples suggested that this variant might have occurred de novo. *COL4A1* encodes the α1 chain of type IV collagen and defects in this gene have been linked to a multisystem disorder that is generally characterized by the presence of cerebrovascular disease with variable ocular, renal, and muscular involvement [MIM 175780].^18^ Cerebrovascular manifestations occur from fetal life onward and their severity may range from small vessel brain disease to fatal cerebral hemorrhage.^19^ Ocular abnormalities are variably observed and may include pediatric cataract, ASD, or retinal arterial tortuosity.^20^ Notably, a family with isolated pediatric cataract has been reported.^21^ Kidney disease (hematuria and renal cysts of variable severity) and painful muscle cramps occur in subsets of affected individuals.^18^ Referrals were made to establish the extent of disease in proband 18014933; no developmental delay, cardiac abnormalities, seizures, or other extraocular features were present as of age 1.5 years.

Proband 17028507 was diagnosed with oculocutaneous albinism at 4 months of age. He presented with nystagmus, iris transillumination, fundal hypopigmentation, and foveal hypoplasia. There was moderate amount of skin and hair pigmentation, there were no other concerns about his health or development, and there was no family history of albinism or visual problems. Genetic testing was requested at 4 months of age revealing a c.1507G>T p.(Glu503Ter) variant in *HPS5* in homozygous state. *HPS5* encodes a protein that has a role in the intracellular trafficking of proteins.^22^ Defects in this gene have been found to cause Hermansky–Pudlak syndrome (HPS), a multisystem disorder characterized by albinism and bleeding diathesis. Granulomatous colitis and pulmonary fibrosis can also be features of this condition although *HPS5* is associated with a less severe form of HPS with only mild bleeding tendency and no gastrointestinal or pulmonary symptoms [MIM 614074].^23^ Proband 17028507 was referred to hematology and platelet function analysis was found to be abnormal: there was prolonged Platelet Function Analyzer-100 (PFA-100) closure time following stimulation with collagen and epinephrine (>300 seconds; reference range 79–205 seconds). This finding had significant implications as the child was due to have a routine urological procedure (orchidopexy) that was safely performed with appropriate precautions (including oral transexamic acid commenced 24 hours prior to the procedure and continued for 5 days, intravenous desmopressin acetate [DDAVP] perioperatively, and HLA matched platelets available to be used in the event of unexpected bleeding).

## DISCUSSION

In this study, we provide evidence in support of routine genetic test provision for specific inherited eye disorders (bilateral pediatric cataracts, bilateral ectopia lentis, bilateral ASD, albinism, and IRD) in children aged 0 to 5 years. The diagnostic yield of panel-based genetic testing in this cohort of 201 probands was 64% (ranging from 39% to 91% depending on the condition; Table 1) and the test result led to altered management in significant subsets of children (ranging from 3% to 75% depending on the condition; Table 3).

The diagnostic rates in this cohort of infants and young children were compared with previous studies with less strict inclusion criteria. For bilateral pediatric cataracts we identified a probable molecular diagnosis in 50% of cases; this is one of the largest cohorts of individuals with this phenotype reported to date (*n* = 74; Table 1). Two previous studies on congenital cataract from Australia reported diagnostic yields of 62% (*n* > 33)^24^ and 68% (*n* = 46);^25^ a study from Saudi Arabia reported a diagnostic yield of 58% (*n* = 74)^26^ and five further studies on Chinese cohorts reported diagnostic yields of 26% (*n* = 74),^27^ 59% (*n* = 39),^28^ 62% (*n* = 21),^29^ 63% (*n* = 27),^30^ and 68% (*n* = 34).^31^ For bilateral ectopia lentis we identified a probable molecular diagnosis in 75% of a relatively small cohort (*n* = 8; Table 1). Previous studies from the Netherlands, the UK, and China reported diagnostic yields of 67% (*n* = 24),^32^ 72% (*n* = 18),^6^ and 85% (*n* = 40)^33^ respectively. In children with bilateral ASD, we identified a probable molecular diagnosis in 39% of cases (*n* = 28; 16/28 only had glaucoma with no other features of ASD—the yield for this subset was 31%; Table 1). A recent UK study of children with this clinical presentation reported a diagnostic yield of 25% (*n* = 113; 60/113 only had glaucoma with no other features of ASD—the yield for this subset was 13%).^34^ For albinism, we identified a probable molecular diagnosis in 91% of cases (*n* = 32; Table 1); a large study from France summarizing data from 990 probands with albinism reported a diagnostic yield of 72% (*n* = 990).^4^ For IRD, we identified a probable molecular diagnosis in 78% of cases (*n* = 59; Table 1). A previous study on pediatric IRD from our group reported a diagnostic yield of 79% (*n* = 85)^2^ while a landmark study on 1000 patients with IRD (aged ≤60 years) reported a diagnostic yield of 76% (*n* = 1000).^7^ Also, a recent study reporting findings from 1243 IRD patients of Chinese origin reported a diagnostic yield of 72% (*n* = 1243).^35^ Overall, these comparisons suggest that the likelihood of a genetic test identifying a molecular diagnosis are linked to (1) the current state of knowledge of the genetic architecture of the patient’s condition (e.g., higher for albinism and IRD than for cataract and ASD); (2) the pretest probability of the patient having a penetrant monogenic disorder (typically higher for bilateral disease, when there is relevant family history, for specific well-recognized clinical presentations and/or for early childhood onset conditions).^35,36^

Like any other medical interventions, diagnostic genetic tests should be thoroughly evaluated before their introduction into routine practice. Increasingly, decision makers request more than measures of a test’s technical performance and diagnostic yield; clinical utility has therefore emerged as a key concept.^9,10^ Clinical utility is contextual and we have studied different clinical scenarios in a population of children aged 0 to 5 years.

For bilateral congenital cataract, previous studies have highlighted how genetic testing can help identify clinically unrecognized syndromic associations (e.g., when the clinical signs are subtle or still evolving); examples include oculofaciocardiodental syndrome (*BCOR*-related disease [MIM 300166]),^25,28^ Nance–Horan syndrome [MIM 302350],^25,31^ and certain metabolic conditions (some of which are treatable).^37^ In this study, genetic testing pointed to a previously unsuspected syndrome in 12% (9/74) of cases (Table 3; Supplementary Table 1). Intriguingly, 4 of these 9 probands had *COL4A1*-related disorders [MIM 175780] and were therefore at risk of small vessel brain disease.^20^ Another important finding was that additional tests were avoided in 50% (37/74) of cataract probands: since a molecular diagnosis was identified in these children there was no need for further diagnostic work-up. It is noteworthy that clinical utility cannot be defined in an absolute sense and has to be evaluated relative to a comparator strategy.^10^ Traditionally, children with bilateral cataracts without a family history had a number of investigations (including chromosomal analysis, extensive biochemical tests, and tests looking for evidence of intrauterine infection).^3^ We have previously reported that genetic analysis has significant advantages compared with this traditional approach,^38^ suggesting that the above clinical utility estimate is conservative. Overall, we provide further evidence supporting a central role of genetic testing to the care pathway of children with cataract.

For ectopia lentis, genetic testing is particularly helpful in confirming or excluding *FBN1*-associated disorders (including Marfan syndrome). In this cohort, 2/8 children were initially diagnosed with apparently isolated ectopia lentis but were found to carry *FBN1* variants and thus benefited from regular surveillance for aortic root disease. In contrast, 4/8 children had a different molecular diagnosis (recessive *ADAMTSL4*- or recessive *LTBP2*-related disease [MIM 225100, 225200 and 251750]; Supplementary Table 1) and did not require extensive systemic investigations. Our findings are in keeping with previous studies and highlight that recessive “isolated” ectopia lentis is a frequent cause and an important differential for this clinical presentation in children.^6,32^

ASD encompasses a variety of clinical presentations^5^ and the clinical utility of genetic testing within this diagnostic category is not uniform. For suspected pediatric primary glaucoma, genetic analysis can help exclude secondary causes. Interestingly, two infants from this cohort who were initially diagnosed with primary glaucoma were found to have a secondary mechanism associated with either *LTBP2* or *FOXC1*^5^ gene defects. Furthermore, one infant who presented with apparently isolated primary glaucoma was found to have Charcot–Marie–Tooth hereditary neuropathy (type 4B2 [MIM 604563]) (Supplementary Table 1). For ASD without glaucoma, there is a growing body of evidence suggesting that the glaucoma risk differs among different genetic causes.^39^ For aniridia, genetic testing is particularly important as it allows early detection/exclusion of two molecular subtypes (accounting for ~10% of aniridia cases) that are associated with life limiting complications: WAGR syndrome [MIM 194072] and *ACTA2*-related multisystemic smooth muscle dysfunction [MIM 613834].^40^

For albinism, it is known that syndromic forms of the disease (HPS and Chediak–Higashi syndrome [MIM 214500]) can be overlooked, especially in young children. Affected individuals may therefore remain undiagnosed until later in life unless genetic testing is performed; this is concerning as these patients require specific management to prevent life limiting complications.^4^ In this study we found that 3% (1/32) of children with presumed nonsyndromic albinism had HPS (proband 17028507, discussed in the “Results” section). Although we identified just a single case, our findings are in general in agreement with a large study in the French population that reported syndromic forms in 5% of all albinism cases (46/990 HPS, 2/990 Chediak–Higashi syndrome [MIM 214500]).^4^ Another finding in the present study is that genetic testing helped refine the diagnosis in three males who were clinically diagnosed with “ocular albinism” (Supplementary Table 1). This clinical presentation may be due to X-linked ocular albinism (*GPR143* gene defects [MIM 300500]), hypomorphic variants in genes associated with oculocutaneous albinism, variants in *SLC38A8* (causing foveal hypoplasia and optic nerve decussation defects [MIM 609218]), or mild forms of HPS (e.g., HPS3 [MIM 614072], HPS5 [MIM 614074] and HPS6 [MIM 614075]).^4^ Of these three male probands, one had X-linked ocular albinism [MIM 300500] and two had partial oculocutaneous albinism. This result led to tailored advice on skin photoprotection and on the risk for developing nonmelanoma skin cancer.

For IRD, a key driver for genetic testing is the advent of gene-directed interventions including gene augmentation therapies and antisense oligonucleotide treatments.^41^ Clinical trials for multiple IRD subtypes are in progress and genetic analysis is required to identify those for whom these treatments might be relevant. Furthermore, IRD can be one of the first presenting features of a syndromic condition such as a ciliopathy (e.g., Bardet–Biedl, Alström [MIM 203800], or Senior-Løken syndrome) or a neurometabolic disorder (e.g., Refsum [MIM 266500] or Batten [MIM 204200] disease). In this cohort, 6% (3/48) of probands had a clinical diagnosis of nonsyndromic IRD but were found to have defects in *CEP290*, a ciliopathy gene that is often associated with extraocular manifestations.^42^ Referrals for further investigations were initiated revealing no relevant systemic manifestations to date. Notably, two previous studies reported that genetic analysis revealed the presence of a syndromic disorder in 1% (3/218)^13^ and 2% (2/85)^2^ of unselected IRD patients (all these 5 cases were initially thought to have nonsyndromic disease). In addition to highlighting the possibility of extraocular involvement, genetic testing can help differentiate progressive from stationary IRD subtypes.^2^ Here, we found that genetic testing helped reduce prognostic uncertainty in 25% (15/59) of study participants. Interestingly, approximately 1 in 4 children in whom a progressive disorder was strongly considered were shown to have a stationary condition (Supplementary Table 1).

There are some important caveats that are worth highlighting. First, the utility of genetic testing has different dimensions (public health, clinical, personal, societal) and focusing only on management change is insufficient. We believe that there is great value in establishing a molecular diagnosis even when there is no direct impact on management. A precise diagnosis can not only lead to early resolution of uncertainty and better understanding of the condition but it also can inform reproductive and life planning. However, outcomes such as well-being, quality of life, and impact on family members have a degree of subjectivity that makes devising appropriate measures and research designs challenging.^10^ Second, the ability of a positive test result to inform clinical practice is only one aspect of clinical utility. A more complete assessment would involve evaluating the balance of health benefits and potential harms that accrue from positive and negative results. A positive test result could lead to anxiety, “overdiagnosis,” and unnecessary treatment while a negative result can make the tested individual feel disappointed and demotivated. To minimize these risks, careful pretest counseling and a narrow pretest clinical hypothesis (that reduces the false discovery rate)^7^ are required. Third, it is worth noting that clinical utility is typically based on the utility of the intervention guided by the test, rather than the test itself (e.g., excision of a Wilms tumor following a genetic test for aniridia). However, the evidence for many such interventions is not robust (e.g., β-blocker initiation to prevent aortic dissection in patients with Marfan syndrome). There is a need for well-defined protocols that link test results to specific clinical responses and downstream management (e.g., screening protocols for renal disease in individuals with defects in ciliopathy genes). These protocols should be based on current best evidence and the generated data should be used as a basis for further evidence generation. Finally, we note that the ideal study design for assessing clinical utility is a randomized controlled trial (as this design maximizes internal validity and addresses issues of selection bias and confounding). However, for each test, the required level of evidence depends on the clinical indication and setting. Although this retrospective observational study is experimentally less rigorous compared with a prospective randomized trial, it allowed collection of data on long term outcomes and it is arguably more representative of real-world clinical care (making its results more likely to be generalizable).

In summary, this study highlights the potential of genetic testing to deliver a precise diagnosis with a high success rate in infants and young children suspected to have an inherited eye disorder. Genetic testing can point to mild, unrecognized, or previously disregarded syndromic features. This often leads to downstream clinical management that can have important implications for the child’s health and development. Our findings provide further evidence in support of the use of genetic testing as a frontline diagnostic tool for bilateral pediatric cataracts, bilateral ectopia lentis, bilateral ASD, albinism, and IRD.

## Supplementary information

Supplementary Tables
