# Supplementary material for: Clinical utility of genetic testing in 201 preschool children with inherited eye disorders
Source: Genet Med. 2019 Dec 18;22(4):745–51. doi: 10.1038/s41436-019-0722-8 (PMC7118019; doi:10.1038/s41436-019-0722-8)
Supplement: Supplementary file 1 — Supplementary Tables [file 41436_2019_722_MOESM1_ESM.pdf]

Supplementary Information

Clinical utility of genetic testing in 201 pre-school children with inherited eye disorders

Eva Lenassi, PhD, FEBO[1,2]; Jill Clayton-Smith, MD, FRCP[1,2]; Sofia Douzgou, PhD, FRCP[1,2]; Simon C. Ramsden, PhD, FRCPath[1,2]; Stuart Ingram, MSc[2]; Georgina Hall, MSc[1,2]; Claire L. Hardcastle, BSc[2]; Tracy A. Fletcher, BSc[2]; Rachel L. Taylor, PhD[1,2]; Jamie M. Ellingford, PhD[1,2]; William D. Newman, FRCOphth[3]; Cecilia Fenerty, FRCOphth[1,2]; Vinod Sharma, FRCOphth[3]; I. Chris Lloyd, FRCOphth[1,4]; Susmito Biswas, FRCOphth[1,3]; Jane L. Ashworth, PhD, FRCOphth[1,3]; Graeme C. Black, DPhil, FRCOphth[1,2]; Panagiotis I. Sergouniotis, PhD, FRCOphth[1,2,3]

[1] Division of Evolution and Genomic Sciences, School of Biological Sciences, Faculty of Biology, Medicines and Health, University of Manchester, Manchester, UK.

[2] Manchester Centre for Genomic Medicine, Saint Mary's Hospital, Manchester University NHS Foundation Trust, Manchester, UK.

[3] Manchester Royal Eye Hospital, Manchester University NHS Foundation Trust, Manchester, UK.

[4] Great Ormond Street Hospital for Children NHS Foundation Trust, London, UK

Supplementary Table 1: Clinical and genetic characteristics of the 201 pre-school children included in this study.

labno	gender	age at referral for genetic testing in years	referral diagnosis	relevant family history	number of genes tested	genotype	ACMG variant classification ^	avoiding unneccessary tests	initiating surveillance for extraocular manifestations	reducing prognostic uncertainty	determining eligibility for treatment trials	other / comment	case previously reported by our group
albinism (n=32)													
17001518	male	<1	albinism (oculocutaneous)	no	18	TYR c.1118C>A p.(Thr373Lys) het;<br>TYR c.1217C>T p.(Pro406Leu) het	class 5 (pathogenic);<br>class 5 (pathogenic)						
17028077	male	1	albinism (oculocutaneous)	no	18	TYR c.938C>G p.(Pro313Arg) hom	class 4 (likely pathogenic)					family interested in pre-implantation genetic diagnosis	
17022857	female	<1	albinism (oculocutaneous)	yes	18	TYR c.823G>T p.(Val275Phe) het;<br>TYR c.1264C>T p.(Arg422Trp) het	class 4 (likely pathogenic);<br>class 4 (likely pathogenic)						
16000955	female	3	albinism (oculocutaneous)	no	18	TYR c.1146C>A p.(Asn382Lys) het;<br>TYR c.1217C>T p.(Pro406Leu) het	class 4 (likely pathogenic);<br>class 5 (pathogenic)						
18004390	male	<1	albinism (oculocutaneous)	no	18	TYR c.1146C>A p.(Asn382Lys) het;<br>TYR c.1336G>A p.(Gly446Ser) het	class 4 (likely pathogenic);<br>class 4 (likely pathogenic)					familial concerns about Hermansky-Pudlak syndrome	
16009407	male	3	albinism (oculocutaneous)	no	18	TYR c.107G>A p.(Cys36Tyr) het;<br>TYR c.613C>A p.(Pro205Thr) het	class 3 (VOUS);<br>class 3 (VOUS)					familial concerns about Hermansky-Pudlak syndrome	
16010606	female	<1	albinism (oculocutaneous)	no	18	TYR c.1099C>T p.(His367Tyr) het;<br>TYR c.1118C>A p.(Thr373Lys) het	class 4 (likely pathogenic);<br>class 5 (pathogenic)						
17003996	female	3	albinism (oculocutaneous)	no	18	TYR c.229C>T p.(Arg77Trp) het;<br>TYR c.1204C>T p.(Arg402Ter) het	class 4 (likely pathogenic);<br>class 5 (pathogenic)					familial concerns about Hermansky-Pudlak syndrome	
17001519	male	4	albinism (oculocutaneous);	no	18	TYR c.1114G>A p.(Gly372Arg) hom	class 4 (likely pathogenic)						
16026544	female	<1	albinism (oculocutaneous)	no	18	TYR c.1118C>A p.(Thr373Lys) het;<br>TYR c.575C>A p.(Ser192Tyr) hom;<br>TYR c.1205G>A p.(Arg402Gln) het	class 5 (pathogenic);<br>class 3 (VOUS);<br>class 3 (VOUS)						
17012896	female	1	albinism (oculocutaneous)	no	18	TYR c.242C>T p.(Pro81Leu) het;<br>TYR c.575C>A p.(Ser192Tyr) hom;<br>TYR c.1205G>A p.(Arg402Gln) het	class 3 (VOUS);<br>class 3 (VOUS);<br>class 3 (VOUS)						
17029046	male	1	albinism (oculocutaneous)	no	18	TYR c.1099C>T p.(His367Tyr) het;<br>TYR c.575C>A p.(Ser192Tyr) hom;<br>TYR c.1205G>A p.(Arg402Gln) het	class 4 (likely pathogenic);<br>class 3 (VOUS);<br>class 3 (VOUS)						
16002468	male	4	albinism (oculocutaneous)	affected sister	18	TYR c.1467dupT p.(Ala490CysfsTer20) het;<br>TYR c.575C>A p.(Ser192Tyr) het;<br>TYR c.1205G>A p.(Arg402Gln) het	class 4 (likely pathogenic);<br>class 3 (VOUS);<br>class 3 (VOUS)					familial concerns about Hermansky-Pudlak syndrome	
18017244	male	1	albinism (oculocutaneous)	no	18	TYR c.61C>T p.(Pro21Ser) het;<br>TYR c.575C>A p.(Ser192Tyr) het;<br>TYR c.1205G>A p.(Arg402Gln) het	class 4 (likely pathogenic);<br>class 3 (VOUS);<br>class 3 (VOUS)						
17007832	female	4	albinism (oculocutaneous)	no	18	TYR c.1204C>T p.(Arg402Ter) het;<br>TYR c.575C>A p.(Ser192Tyr) het;<br>TYR c.1205G>A p.(Arg402Gln) het	class 5 (pathogenic);<br>class 3 (VOUS);<br>class 3 (VOUS)						
18006521	female	3	albinism (oculocutaneous)	no	18	TYRP1 c.1103delA p.(Lys368SerfsTer17) het;<br>TYRP1 ex1 to ex5 three copies	class 4 (likely pathogenic);<br>class 4 (likely pathogenic)						
16010468	female	1	albinism (oculocutaneous)	no	18	TYRP1 c.725_728dupCTTC p.(Ser245PhefsTer16) het;<br>TYRP1 c.395A>T p.(Asn132Ile) het	class 5 (pathogenic);<br>class 3 (VOUS)						

labno	gender	age at referral for genetic testing in years	referral diagnosis	relevant family history	number of genes tested	genotype	ACMG variant classification	avoiding unneccessary tests	initiating surveillance for extraocular manifestations	reducing prognostic uncertainty	determining eligibility for treatment trials	other / comment	case previously reported by our group
16014555	male	2	albinism (oculocutaneous)	no	18	OCA2 c.2020C>G p.(Leu674Val) hom	class 3 (VOUS)						
16021414	male	4	albinism (oculocutaneous)	affected sister	18	OCA2 c.1327G>A p.(Val443Ile) hom	class 4 (likely pathogenic)						
16023117	male	<1	albinism (oculocutaneous)	no	18	OCA2 exon 7 deletion hom	class 5 (pathogenic)						
17018603	male	4	albinism (oculocutaneous)	younger brother affected	18	OCA2 exon 7 deletion hom	class 5 (pathogenic)					familial concerns about Hermansky-Pudlak syndrome	
17017561	male	<1	albinism (oculocutaneous)	no	18	OCA2 c.1664G>C p.(Arg555Pro) het;<br>OCA2 c.1841dupA p.(His615AlafsTer2) het	class 3 (VOUS);<br>class 5 (pathogenic)						
18004387	female	<1	albinism (oculocutaneous)	no	18	OCA2 c.1951+1G>A het;<br>OCA2 c.2330G>A p.(Cys777Tyr) het	class 5 (pathogenic);<br>class 3 (VOUS)						
18004262	female	<1	albinism (oculocutaneous)	no	18	OCA2 c.2051_2052delinsG p.(Phe684CysfsTer8) het	class 5 (pathogenic)					familial concerns about Hermansky-Pudlak syndrome	
18012132	female	<1	albinism (oculocutaneous)	no	26	OCA2 c.1327G>A p.(Val443Ile) het<br>OCA2 exons 19 to 21 duplication het	class 4 (likely pathogenic);<br>class 3 (VOUS)						
17028507	male	<1	albinism (oculocutaneous)	no	18	HPS5 c.1507G>T p.(Glu503Ter) hom	class 5 (pathogenic)		haematology referral initiated				
16010469	male	1	albinism (oculocutaneous)	no	18	no clearly causative variant identified	not applicable					phentype unlike classical albinism	
17013894	male	<1	albinism (ocular or oculocutaneous)	no	18	GPR143 c.659-1G>A hemi	class 5 (pathogenic)					tailored advice on skin photoprotection	yes [PMID:31719542]
16017162	male	1	albinism (ocular or oculocutaneous)	yes	40	TYR c.1118C>A p.(Thr373Lys) het;<br>TYR c.1205G>A p.(Arg402Gln) het;<br>TYR c.575C>A p.(Ser192Tyr) hom	class 5 (pathogenic);<br>class 3 (VOUS);<br>class 3 (VOUS)					tailored advice on skin photoprotection	yes [PMID:31719542]
17011637	male	1	albinism (ocular or oculocutaneous)	no	18	TYR c.1217C>T p.(Pro406Leu) het;<br>TYR c.1205G>A p.(Arg402Gln) het;<br>TYR c.575C>A p.(Ser192Tyr) het	class 5 (pathogenic);<br>class 3 (VOUS);<br>class 3 (VOUS)					tailored advice on skin photoprotection	yes [PMID:31719542]
16006232	male	<1	albinism (oculocutaneous);<br>developmental delay;<br>epileptic encephalopathy	yes	18	TYR c.38delT p.(Phe13SerfsTer18) hom	class 5 (pathogenic)						
17022050	male	5	albinism (ocular);<br>developmental delay;<br>microcephaly	no	18	no clearly causative variant identified	not applicable						yes [PMID:31719542]

↓

labno	gender	age at referral for genetic testing in years	referral diagnosis	relevant family history	number of genes tested	genotype	ACMG variant classification	avoiding unneccessary tests	initiating surveillance for extraocular manifestations	reducing prognostic uncertainty	determining eligibility for treatment trials	other / comment	case previously reported by our group
bilateral paediatric cataract (n=74)													
17026278	male	<1	congenital cataracts	yes	144	CRYAA c.61C>T p.(Arg21Trp) het	class 4 (likely pathogenic)	no further diagnostic work-up required					
17021643	male	<1	congenital cataracts; microphthalmia	no	144	CRYAA c.34C>T p.(Arg12Cys) het	class 4 (likely pathogenic)	no further diagnostic work-up required					
15007971	female	<1	congenital cataracts	yes	114	CRYAA c.62G>T p.(Arg21Leu) het	class 4 (likely pathogenic)	no further diagnostic work-up required					
15021428	male	<1	congenital cataracts	yes	114	CRYAA c.61C>T p.(Arg21Trp) het	class 4 (likely pathogenic)	no further diagnostic work-up required					
14017471	female	1	congenital cataracts	no	114	CRYBA1 c.475G>A p.(Gly159Ser) het	class 4 (likely pathogenic)	no further diagnostic work-up required					
15016254	female	5	congenital cataracts	no	114	CRYBA1 c.215+2T>C het	class 3 (VOUS)	no further diagnostic work-up required					
16026011	male	1	congenital cataracts	yes	144	CRYBA1 c.215+2T>A het	class 4 (likely pathogenic)	no further diagnostic work-up required					
17030224	female	<1	congenital cataracts; trisomy 21	no	144	CRYBB1 c.473dupA p.(Asn158LysfsTer24) het	class 3 (VOUS)	no further diagnostic work-up required					
15010948	female	<1	congenital cataracts	no	114	CRYGD c.127T>C p.(Trp43Arg) het	class 4 (likely pathogenic)	no further diagnostic work-up required					
15004858	female	4	congenital cataracts	no	114	CRYAB c.295G>T p.(Glu99Ter) het	class 4 (likely pathogenic)	no further diagnostic work-up required	cardiology referral initiated				
15019910	female	5	congenital cataracts	yes	114	GJA3 c.148T>C p.(Ser50Pro) het	class 4 (likely pathogenic)	no further diagnostic work-up required					yes [PMID: 25148791]
17008393	male	3	congenital cataracts	yes	144	GJA3 c.148T>C p.(Ser50Pro) het	class 4 (likely pathogenic)	no further diagnostic work-up required					
15018151	female	<1	congenital cataracts	no	114	GJA8 c.263C>T p.(Pro88Leu) het	class 3 (VOUS)	no further diagnostic work-up required					
11012212	female	<1	congenital cataract	yes	144	GJA8 c.293A>G p.(His98Arg) het	class 3 (VOUS)	no further diagnostic work-up required					yes [PMID: 25148791]
18009736	male	2	congenital cataracts	yes	144	BFSP2 c.697_699del p.(Glu233del) het	class 4 (likely pathogenic)	no further diagnostic work-up required					yes [PMID: 25148791]
14011092	female	<1	congenital cataracts	no	114	chromosome deletion involving whole BCOR gene het	class 3 (VOUS)	no further diagnostic work-up required	cardiology referral initiated				
15014851	female	<1	congenital cataracts	no	114	HSF4 c.479T>G p.(Leu160Arg) hom	class 3 (VOUS)	no further diagnostic work-up required					

labno	gender	age at referral for genetic testing in years	referral diagnosis	relevant family history	number of genes tested	genotype	ACMG variant classification	avoiding unneccessary tests	initiating surveillance for extraocular manifestations	reducing prognostic uncertainty	determining eligibility for treatment trials	other / comment	case previously reported by our group
17003685	female	<1	congenital cataracts	no	144	HSF4 c.503G>A p.(Trp168Ter) het; HSF4 c.1188+1G>A het	class 5 (pathogenic); class 5 (pathogenic)	no further diagnostic work-up required					
18010355	male	<1	congenital cataracts	no	144	FYCO1 c.2443delA p.(Met815TrpfsTer6) het; FYCO1 c.1237delG p.(Glu413ArgfsTer25) het	class 5 (pathogenic); class 5 (pathogenic)	no further diagnostic work-up required					
15016572	female	<1	congenital cataracts	no	114	FYCO1 c.539+3A>G het; FYCO1 c.395+2T>G het	class 4 (likely pathogenic); class 4 (likely pathogenic)	no further diagnostic work-up required					
15021966	male	<1	congenital cataracts	yes	114	PITX3 c.640_656del p.(Ala214ArgfsTer42) het	class 5 (pathogenic)	no further diagnostic work-up required					
17015905	male	5	congenital cataracts	yes	144	PITX3 c.640_656dup p.(Gly220ProfsTer95) het	class 5 (pathogenic)	no further diagnostic work-up required					
16017070	male	<1	congenital cataracts	no	114	FOXE3 c.352A>G p.(Ser118Gly) hom	class 3 (VOUS)	no further diagnostic work-up required					
16001640	male	2	congenital cataracts	no	114	MIP c.559C>T p.(Arg187Cys) het; CRYAA c.410G>C p.(Gly137Ala) het	class 4 (likely pathogenic); class 3 (VOUS)	no further diagnostic work-up required					
17004217	male	<1	congenital cataracts; trisomy 21	no	144	VIM c.1080C>G p.(Asp360Glu) het	class 3 (VOUS)	no further diagnostic work-up required					
15000185	male	<1	congenital cataracts; trisomy 21	no	114	FOXE3 c.431A>G, p.(Tyr144Cys) het	class 3 (VOUS)	no further diagnostic work-up required					
16022871	male	5	congenital cataracts	no	144	LONP1 c.1661A>T p.(Asp554Val) hom	class 3 (VOUS)	no further diagnostic work-up required					
14022259	male	<1	congenital cataracts	no	114	NHS c.3127C>T p.(Gln1043Ter) hemi *[de novo]*	class 5 (pathogenic)	no further diagnostic work-up required	dental referral initiated; development closely				
17014480	female	<1	congenital cataracts; microphthalmia	yes	144	RAB3GAP1 c.1417_1423del p.(Gly473LysfsTer23) hom	class 5 (pathogenic)	no further diagnostic work-up required	development closely monitored			family interested in pre-implantation genetic diagnosis	
16024006	male	<1	congenital cataracts; microphthalmia	no	144	COL4A1 c.2317G>A p.(Gly773Arg) het	class 4 (likely pathogenic)	no further diagnostic work-up required	cardiology referral & renal screening initiated				
18014933	female	<1	congenital cataracts	no	144	COL4A1 c.2317G>A p.(Gly773Arg) het *[de novo]*	class 4 (likely pathogenic)	no further diagnostic work-up required	cardiology referral & renal screening initiated			result informed reproductive planning	
14001458	male	<1	congenital cataracts; cerebral infarction	no	114	COL4A1 c.2273G>A p.(Gly758Glu) het *[de novo]*	class 4 (likely pathogenic)	no further diagnostic work-up required	renal screening initiated			result informed reproductive planning	
15000884	male	3	congenital cataracts; developmental delay; neonatal poroncephalic cyst	no	114	COL4A1 c.2662G>C p.(Gly888Arg) het *[de novo]*	class 4 (likely pathogenic)	no further diagnostic work-up required	renal screening initiated			result informed reproductive planning	
14022248	male	2	congenital cataracts; developmental delay; cerebellar atrophy	no	genome sequencing	SIL1 exon 3 to 4 deletion hom	class 5 (pathogenic)	no further diagnostic work-up required					
14013821	female	<1	congenital cataracts; microphthalmia; microcephaly; developmental delay	no	genome sequencing	CTNNB1 c.884C>G p.(Ala295Gly) hom	class 3 (VOUS)	no further diagnostic work-up required					

labno	gender	age at referral for genetic testing in years	referral diagnosis	relevant family history	number of genes tested	genotype	ACMG variant classification	avoiding unneccessary tests	initiating surveillance for extraocular manifestations	reducing prognostic uncertainty	determining eligibility for treatment trials	other / comment	case previously reported by our group
13006157	female	<1	congenital cataracts; developmental delay; congenital hypothyroidism; subependymal nodules	no	genome sequencing	NALCN c.1186G>A p.(Val396Met) het *[de novo]*	class 3 (VOUS)	no further diagnostic work-up required				result informed reproductive planning	
18018483	female	2	congenital cataract; developmental delay; lumbar spine skin dimple	no	144	GALK1 c.998T>C p.(Leu333Pro) hom	class 3 (VOUS)	no further diagnostic work-up required	metabolic work-up initiated				
16014046	male	<1	congenital cataracts	no	114	no clearly causative variant identified; CRYAB c.460G>A p.(Gly154Ser) het	class 3 (VOUS)						
15004571	female	<1	congenital cataracts	no	114	no clearly causative variant identified; ADAMTSL4 c.767_768del het; ADAMTSL4 c.2520dupC het	class 5 (pathogenic); class 5 (pathogenic)						
15001368	female	5	congenital cataracts	yes	114	no clearly causative variant identified; COL2A1 c.4148C>T p.(Thr1383Met) het	class 3 (VOUS)						
17013599	male	1	congenital cataracts	yes	144	no clearly causative variant identified; RAB3GAP2 c.4175_4178del p.(Ser1392TyrfsTer10) het	class 3 (VOUS)						
17001526	female	1	congenital cataracts	no	144	no clearly causative variant identified; CBS c.1105C>T p.(Arg369Cys) het	class 4 (likely pathogenic)						
17001521	female	5	congenital cataracts	no	144	no clearly causative variant identified; ALDH18A1 c.1273C>T p.(Arg425Cys) het	class 3 (VOUS)						
16004017	male	3	congenital cataracts	no	114	no clearly causative variant identified	not applicable						
16013132	female	3	congenital cataracts	no	114	no clearly causative variant identified	not applicable						
15003590	male	4	congenital cataracts	no	114	no clearly causative variant identified	not applicable						
18009939	male	<1	congenital cataracts	no	144	no clearly causative variant identified	not applicable						
12009672	male	1	congenital cataracts	yes	114	no clearly causative variant identified	not applicable						
14005417	male	1	congenital cataracts	no	114	no clearly causative variant identified	not applicable						
13018264	male	5	congenital cataracts	no	114	no clearly causative variant identified	not applicable						
14016196	male	5	congenital cataracts	no	114	no clearly causative variant identified	not applicable						
14018931	male	4	congenital cataracts	no	114	no clearly causative variant identified	not applicable						
14020759	female	5	congenital cataracts	no	114	no clearly causative variant identified	not applicable						

labno	gender	age at referral for genetic testing in years	referral diagnosis	relevant family history	number of genes tested	genotype	ACMG variant classification	avoiding unneccessary tests	initiating surveillance for extraocular manifestations	reducing prognostic uncertainty	determining eligibility for treatment trials	other / comment	case previously reported by our group
16024573	female	4	congenital cataracts	no	144	no clearly causative variant identified	not applicable						
17000928	female	5	congenital cataracts	no	144	no clearly causative variant identified	not applicable						
14017818	male	<1	congenital cataracts	no	114	no clearly causative variant identified	not applicable						
17027784	male	4	congenital cataracts	no	144	no clearly causative variant identified	not applicable						
18001837	female	4	congenital cataracts	no	144	no clearly causative variant identified	not applicable						
16013003	female	1	congenital cataracts	no	114	no clearly causative variant identified	not applicable						
15015181	male	4	congenital cataracts	no	114	no clearly causative variant identified;<br>ERCC3 c.1757delA p.(Gln586ArgfsTer25) het	 class 5 (pathogenic)						
17026533	male	5	congenital cataracts; posterior lenticonus	yes	144	no clearly causative variant identified;<br>RECQL4 c.2492_2493del p.(His831ArgfsTer52) het	 class 5 (pathogenic)						
15019612	female	<1	congenital cataracts; microcornea; Axenfeld-Rieger anomaly	yes	114	no clearly causative variant identified	not applicable						
15000484	male	4	congenital cataracts; high myopia	no	114	no clearly causative variant identified	not applicable						
17022852	male	<1	congenital cataracts; high myopia; global developmental delay; cerebral palsy	no	144	no clearly causative variant identified	not applicable						
16002025	male	<1	congenital cataracts; unilateral ptosis	no	114	no clearly causative variant identified	not applicable						
14007961	male	4	congenital cataract; congenital glaucoma	no	114	no clearly causative variant identified;<br>COL11A1 c.328G>C p.(Gly110Arg) het	 class 3 (VOUS)						
16016530	female	3	congenital cataracts; developmental delay	no	144	no clearly causative variant identified	not applicable						
18004405	male	3	congenital cataracts; developmental delay	no	144	no clearly causative variant identified	not applicable						
15004516	male	2	congenital cataracts; developmental delay; microcephaly; infantile spasms	no	114	no clearly causative variant identified	not applicable						
12003591	male	2	congenital cataracts; developmental delay; microcephaly; hypospadias	no	114	no clearly causative variant identified;<br>GALK1 c.997C>T p.(Leu333Trp) het	 class 4 (likely pathogenic)					metabolic work-up negative	
16015230	female	1	congenital cataracts; developmental delay; poydactyly; periventricular polymicrogyria	no	114	no clearly causative variant identified	not applicable						

labno	gender	age at referral for genetic testing in years	referral diagnosis	relevant family history	number of genes tested	genotype	ACMG variant classification	avoiding unneccessary tests	initiating surveillance for extraocular manifestations	reducing prognostic uncertainty	determining eligibility for treatment trials	other / comment	case previously reported by our group
14003960	male	4	congenital cataracts; microcephaly; neonatal asphyxia	no	genome sequencing	no clearly causative variant identified	not applicable						
14018514	male	4	congenital cataracts; sparse scalp hair; narrow nasal passages	no	114	no clearly causative variant identified	not applicable						
15019481	female	4	congenital cataract; cerebral palsy; poroncephaly	no	114	no clearly causative variant identified	not applicable						
**bilateral ectopia lentis (n=8)**													
15022427.72	female	3	ectopia lentis	no	144	LTBP2 c.507C>G p.(Cys169Trp) hom	class 3 (VOUS)	extensive systemic investigations not required					
16012251	female	1	ectopia lentis	no	114	LTBP2 c.3427delC p.(Gln1143ArgfsTer35) hom	class 5 (pathogenic)	extensive systemic investigations not required					
17008394	male	3	ectopia lentis	no	144	FBN1 c.356G>A p.(Cys119Tyr) het	class 3 (VOUS)		cardiology referral initiated				
14000821	male	1	ectopia lentis et pupillae	no	114	ADAMTSL4 c.767_786del hom	class 5 (pathogenic)	extensive systemic investigations not required					
13008723	male	5	ectopia lentis	no	114	ADAMTSL4 c.2236C>T p.(Arg746Cys) het; ADAMTSL4 c.767_786del p.(Gln256ProfsTer38) het	class 3 (VOUS); class 5 (pathogenic)	extensive systemic investigations not required					
17001295	female	4	ectopia lentis	no	12	FBN1 c.6354C>T p.(Ile2118Ile) het	class 4 (likely pathogenic)		cardiology referral initiated				
15010654 & 15002428	female	5	ectopia lentis; heart defect	yes	114	no clearly causative variant identified	not applicable						
17003542	male	4	ectopia lentis; joint hypermobility	no	144	no clearly causative variant identified; DHCR7 c.866C>T p.(Thr289Ile) het	class 3 (VOUS)						

↓

labno	gender	age at referral for genetic testing in years	referral diagnosis	relevant family history	number of genes tested	genotype	ACMG variant classification	avoiding unneccessary tests	initiating surveillance for extraocular manifestations	reducing prognostic uncertainty	determining eligibility for treatment trials	other / comment	case previously reported by our group
bilateral anterior segment dysgenesis (ASD) including congenital glaucoma (n=28)													
16025893	male	2	corneal endothelial dystrophy	no	32	ZEB1 c.1440_1441del p.(Gln481AlafsTer2) het	class 5 (pathogenic)						
13013898	male	<1	megalocornea;<br>glaucoma	no	114	LTBP2 c.3578_3581dup p.(Ser1195Ter) het;<br>LTBP2 c.469delA p.(Thr157ProfsTer123) het	class 5 (pathogenic);<br>class 5 (pathogenic)						
17012891	female	2	aniridia	yes	45	PAX6 c.991C>T p.(Arg331Ter) het	class 5 (pathogenic)	confirmed that risk of WAGR syndrome is low					
17015864	male	<1	anterior segment dysgnenesis	no	45	FOXE3 c.325C>A p.(Arg109Ser) het;<br>FOXE3 c.555dupC p.(Phe186LeufsTer99) het	class 3 (VOUS);<br>class 4 (likely pathogenic)						
17009449	female	<1	anterior segment dysgnenesis	no	45	FOXC1 c.481A>G p.(Met161Val) het	class 4 (likely pathogenic)						
14018019	female	<1	congenital glaucoma;<br>anterior segment dysgenesis;<br>excess umbilical skin	no	114	PITX2 c.429_431del p.(Arg144del) het<br>*[de novo]*	class 4 (likely pathogenic)						
15022193	male	<1	congenital glaucoma	no	45	FOXC1 c.256C>T p.(Leu86Phe) het	class 4 (likely pathogenic)						
15022168	male	<1	congenital glaucoma	no	45	SBF2 c.141+1G>A hom	class 5 (pathogenic)		neurology referral initiated			family interested in pre-implantation genetic diagnosis	
16021601	female	4	congenital glaucoma	no	45	CYP1B1 c.1103G>A p.(Arg368His) het;<br>CYP1B1 c.1159G>A p.(Glu387Lys) het	class 4 (likely pathogenic);<br>class 4 (likely pathogenic)						
16000470	male	<1	congenital glaucoma	yes	45	CYP1B1 c.1330C>T p.(Arg444Ter) hom	class 4 (likely pathogenic)						
18018901	male	4	congenital glaucoma	no	45	CYP1B1 c.1103G>A p.(Arg368His) hom	class 4 (likely pathogenic)						
18007394	male	<1	congenital glaucoma	no	45	CYP1B1 c.241T>A p.(Tyr81Asn) het	class 3 (VOUS)						
16000577	female	3	megalocornea	yes	32	no clearly causative variant identified;<br>COL8A2 c.1466dupC p.(Gly490ArgfsTer49) het	class 3 (VOUS)						
17022167	female	<1	posterior embryotoxa	no	45	no clearly causative variant identified	not applicable						
17023814	male	<1	anterior segment dysgnenesis	no	45	no clearly causative variant identified	not applicable						
17014067	male	3	anterior segment dysgenesis;<br>congenital aphakia; unilateral microphthalmia	no	45	no clearly causative variant identified	not applicable						
16025403	male	5	anterior segment dysgenesis;<br>abnormal teeth	no	45	no clearly causative variant identified	not applicable						

labno	gender	age at referral for genetic testing in years	referral diagnosis	relevant family history	number of genes tested	genotype	ACMG variant classification	avoiding unneccessary tests	initiating surveillance for extraocular manifestations	reducing prognostic uncertainty	determining eligibility for treatment trials	other / comment	case previously reported by our group
11012360	male	3	anterior segment dysgenesis; pyloric stenosis	no	genome sequencing	no clearly causative variant identified	not applicable						
16018095	male	5	congenital glaucoma	no	45	no clearly causative variant identified	not applicable						
17010130	female	4	congenital glaucoma	no	45	no clearly causative variant identified	not applicable						
17022448	female	1	congenital glaucoma	no	45	no clearly causative variant identified	not applicable						
18006601	female	<1	congenital glaucoma	no	45	no clearly causative variant identified	not applicable						
15017091	female	3	congenital glaucoma	no	45	no clearly causative variant identified	not applicable						
16010439	male	<1	congenital glaucoma	no	45	no clearly causative variant identified	not applicable						
15005400	male	<1	congenital glaucoma	no	114	no clearly causative variant identified	not applicable						
17027068	male	<1	congenital glaucoma	no	45	no clearly causative variant identified	not applicable						
16018323	male	<1	congenital glaucoma	no	45	no clearly causative variant identified	not applicable						
16018928	female	<1	congenital glaucoma	yes	45	no clearly causative variant identified	not applicable						

↓

labno	gender	age at referral for genetic testing in years	referral diagnosis	relevant family history	number of genes tested	genotype	ACMG variant classification	avoiding unneccessary tests	initiating surveillance for extraocular manifestations	reducing prognostic uncertainty	determining eligibility for treatment trials	other / comment	case previously reported by our group
inherited retinal disease (n=59)													
18005694	female	5	achromatopsia	no	177	CNGA3 c.829C>T p.(Arg277Cys) hom	class 4 (likely pathogenic)						
15003158	female	5	achromatopsia	no	177	CNGB3 c.904-2A>T hom	class 4 (likely pathogenic)						yes [PMID:28341476]
16025366	female	5	achromatopsia	no	177	CNGB3 c.806T>C p.(Leu269Pro) hom	class 3 (VOUS)						
13000861	male	2	cone/cone-rod dystrophy or achromatopsia	no	105	CNGB3 c.1148delC p.(Thr383IlefsTer13) hom	class 5 (pathogenic)			result suggested that the condition is non-progressive			
14000069	male	3	cone/cone-rod dystrophy or achromatopsia	no	105	CNGB3 c.901C>T p.(Gln301Ter) het;<br>CNGB3 c.1148delC p.(Thr383IlefsTer13) het	class 5 (pathogenic);<br>class 5 (pathogenic)			result suggested that the condition is non-progressive			yes [PMID:28341476]
14012482	female	3	cone/cone-rod dystrophy or achromatopsia	no	177	CNGB3 c.1148delC p.(Thr383IlefsTer13) het;<br>CNGB3 c.190delG p.(Glu64SerfsTer19) het;<br>CDH23 c.3293A>G p.(Asn1098Ser) het;<br>CEP164 c.4228C>T p.(Gln1410Ter) het	class 5 (pathogenic);<br>class 5 (pathogenic);<br>class 3 (VOUS);<br>class 3 (VOUS)			result suggested that the condition is non-progressive			yes [PMID:28341476]
16019712	female	4	cone/cone-rod dystrophy or achromatopsia	no	177	CNGB3 c.806T>C p.(Leu269Pro) hom	class 3 (VOUS)			result suggested that the condition is non-progressive			
15011628	male	4	cone/cone-rod dystrophy or achromatopsia	no	177	GNAT2 c.605G>A p.(Gly202Glu) hom;<br>ABCA4 c.5882G>A p.(Gly1961Glu) het	class 3 (VOUS);<br>class 5 (pathogenic)			result suggested that the condition is non-progressive			yes [PMID:28341476]
18012826	male	2	cone/cone-rod dystrophy or achromatopsia (initially idiopathic infantile nystagmus)	no	177	CEP290 c.1709C>G p.(Ser570Ter) het;<br>CEP290 c.2414T>C p.(Leu805Pro) het	class 5 (pathogenic);<br>class 3 (VOUS)		renal screening initiated	result suggested that the condition is non-progressive			
18012804	female	<1	cone/cone-rod dystrophy	no	177	CNGB3 c.1148delC p.(Thr383IlefsTer13) hom	class 5 (pathogenic)			result suggested that the condition is non-progressive			
15020633	female	4	cone/cone-rod dystrophy	no	177	KCNV2 c.562T>C p.(Trp188Arg) hom	class 3 (VOUS)			result suggested that the condition is non-progressive			
17006564	female	5	cone/cone-rod dystrophy	no	177	PROM1 c.1354dupT p.(Tyr452LeufsTer13) het;<br>PROM1 c.436C>T p.(Arg146Ter) het	class 5 (pathogenic);<br>class 4 (likely pathogenic)						
16023111	male	3	congenital stationary synaptic dysfunction or retinal dystrophy	no	177	CACNA1F c.2131G>T p.(Glu711Ter) hemi	class 5 (pathogenic)			result suggested that the condition is non-progressive			
17002921	male	3	congenital stationary synaptic dysfunction or retinal dystrophy	no	177	CACNA1F c.1561C>T p.(Arg521Cys) hemi	class 3 (VOUS)			result suggested that the condition is non-progressive			
14002269	male	1	congenital stationary synaptic dysfunction or retinal dystrophy	yes	177	CACNA1F c.4301A>G p.(Asn1434Ser) hemi;<br>BBS1 c.1169T>G p.(Met390Arg) het;<br>CHM c.1922_1933dup p.(Asn644_Leu645insGlnSerThrAsn) hemi	class 3 (VOUS);<br>class 5 (pathogenic);<br>class 3 (VOUS)			result suggested that the condition is non-progressive			yes [PMID:28341476]
14017012	male	4	congenital stationary synaptic dysfunction or retinal dystrophy	yes	177	CACNA1F c.3289G>A p.(Asp1097Asn) hemi	class 3 (VOUS)			result suggested that the condition is non-progressive			yes [PMID:28341476]
18011541	male	5	congenital stationary synaptic dysfunction	no	177	CACNA1F c.4093_4095del p.(Asn1365del) hemi	class 3 (VOUS)						

labno	gender	age at referral for genetic testing in years	referral diagnosis	relevant family history	number of genes tested	genotype	ACMG variant classification	avoiding unneccessary tests	initiating surveillance for extraocular manifestations	reducing prognostic uncertainty	determining eligibility for treatment trials	other / comment	case previously reported by our group
18004252	male	3	congenital stationary synaptic dysfunction	yes	177	CACNA1F c.868C>T p.(Arg290Cys) hemi	class 3 (VOUS)						
16004896	male	1	congenital stationary night blindness	yes	177	NYX c.212G>C p.(Arg71Pro) hemi;<br>EYS c.8133_8137del p.(Phe2712CysfsTer33) het	class 3 (VOUS);<br>class 4 (likely pathogenic)						yes [PMID:28341476]
16001150	male	5	congenital stationary night blindness	yes	177	NYX c.85_108del p.(Arg29_Ala36del) hemi	class 5 (pathogenic)						yes [PMID:28341476]
15015157	male	4	congenital stationary night blindness	no	177	RHO c.328T>C p.(Cys110Arg) het	class 4 (likely pathogenic)						yes [PMID:28341476]
11010142	male	6	retinal dystrophy	no	177	NYX c.106_111del p.(Ala36_Cys37del) hemi	class 4 (likely pathogenic)			result suggested that condition is non-progressive			yes [PMID:28341476]
15011865	female	<1	retinal dystrophy	no	177	CNGA3 c.667C>T p.(Arg223Trp) het<br>CNGA3 c.1279C>T p.(Arg427Cys) het	class 3 (VOUS);<br>class 4 (likely pathogenic)			result suggested that condition is non-progressive			yes [PMID:28341476]
16009408	male	3	retinal dystrophy	no	177	CABP4 c.673C>T p.(Arg225Ter) hom	class 4 (likely pathogenic)			result suggested that condition is non-progressive			yes [PMID:28341476]
13015227	male	<1	retinal dystrophy	no	105	RPE65 c.726-2A>C hom;<br>CEP290 c.7171_7172del p.(Gln2391AlafsTer26) het	class 5 (pathogenic);<br>class 3 (VOUS)				as per PMID:28712537; formally approved treatment		
17027106	male	3	retinal dystrophy	no	177	RDH12 c.601T>C p.(Cys201Arg) hom	class 5 (pathogenic)						
12013881	female	5	retinal dystrophy	yes	105	RDH12 c.601T>C p.(Cys201Arg) hom;<br>AIPL1 c.465G>T p.(Gln155His) het	class 5 (pathogenic);<br>class 3 (VOUS)						
14003957	female	5	retinal dystrophy	no	105	RDH12 c.506G>A p.(Arg169Gln) het;<br>RDH12 c.883C>T p.(Arg295Ter) het;<br>ABCA4 c.5882G>A p.(Gly1961Glu) het	class 4 (likely pathogenic);<br>class 3 (VOUS);<br>class 5 (pathogenic)						yes [PMID:28341476]
18001788	male	4	retinal dystrophy	no	177	AIPL1 c.834G>A p.(Trp278Ter) het<br>AIPL1 c.[643-5G>A; 784G>A] p.[(?);(Gly262Ser)] het	class 5 (pathogenic);<br>class 3 (VOUS) *in cis* with class 4 (likely pathogenic)						
17016701	male	3	retinal dystrophy	no	177	AIPL1 c.834G>A p.(Trp278Ter) hom	class 5 (pathogenic)						
15014189	male	1	retinal dystrophy	no	177	GUCY2D c.2837C>A p.(Ala946Glu) het;<br>GUCY2D c.2595delG p.(Lys866ArgfsTer14) het	class 3 (VOUS);<br>class 5 (pathogenic)						yes [PMID:28341476]
12007217	male	3	retinal dystrophy	no	105	CEP290 c.2991+1665A>G het;<br>CEP290 c.4966G>T p.(Glu1656Ter) het	Class 5; Pathogenic;<br>Class 5; Pathogenic		renal screening initiated		as per PMID: 30559420		
12004181	male	1	retinal dystrophy	no	105	CEP290 c.451C>T p.(Arg151Ter) het;<br>CEP290 c.3181_3182del het	class 5 (pathogenic);<br>class 5 (pathogenic)		renal screening initiated				
15004850	male	5	retinal dystrophy	no	177	NRL c.479T>C p.(Leu160Pro) hom;<br>CACNA1F c.1903G>A p.(Val635Ile) hemi	class 5 (pathogenic);<br>class 3 (VOUS)						yes [PMID:28341476]
15004859	male	2	retinal dystrophy	no	177	RPGRIP1 c.2079C>G p.(Tyr693Ter) het;<br>RPGRIP1 ex19 del het;<br>ABCA4 c.5196+1136C>A het	class 5 (pathogenic);<br>class 4 (likely pathogenic);<br>class 3 (VOUS)						yes [PMID:28341476]

labno	gender	age at referral for genetic testing in years	referral diagnosis	relevant family history	number of genes tested	genotype	ACMG variant classification	avoiding unneccessary tests	initiating surveillance for extraocular manifestations	reducing prognostic uncertainty	determining eligibility for treatment trials	other / comment	case previously reported by our group
18012273	male	5	retinal dystrophy	no	177	CRB1 c.2536G>A p.(Gly846Arg) hom	class 4 (likely pathogenic)						
14009965	male	3	retinal dystrophy; macular edema	no	105	CRB1 c.584G>T p.(Cys195Phe) het; CRB1 c.2843G>A p.(Cys948Tyr) het	class 4 (likely pathogenic); class 4 (likely pathogenic)						yes [PMID:28341476]
12000202	female	5	retinal dystrophy; hearing impairment	no	105	MYO7A c.[905G>A; 5899C>T] p.[(Arg302His);(Arg1967Ter)] het; MYO7A c.[132+5G>A; 1135G>A] p.[(?);(Gly379Arg)] het;	class 3 (VOUS) *in cis* with class 5 (pathogenic); class 3 (VOUS) in cis with class 3 (VOUS)						
14002664	male	1	retinal dystrophy; polydactyly; micropenis; obesity	no	105	BBS1 c.1339G>A p.(Ala447Thr) hom	class 4 (likely pathogenic)		cardiology referral initiated				yes [PMID:28341476]
13009772	female	<1	retinal dystrophy; polydactyly; recurrent chest infections	no	105	CEP290 c.5668G>T p.(Gly1890Ter) hom; CNGB3 c.1208G>A p.(Arg403Gln) het	class 5 (pathogenic); class 3 (VOUS)		renal screening initiated				
12002598	male	3	retinal dystrophy; learning difficulties; anosmia; obesity	no	105	BBS9 c.1877_1880del p.(Lys626ArgfsTer22) het; IMPDH1 c.189A>G p.(Ser63Ser) het	class 5 (pathogenic); class 3 (VOUS)		renal screening initiated				
16026573	male	2	retinal dystrophy	no	177	GUCY2D c.2837C>A p.(Ala946Glu) het	class 3 (VOUS)						
18007064	female	<1	retinal dystrophy	no	177	no clearly causative variant identified	not applicable						
13016425	male	3	retinal dystrophy	no	105	no clearly causative variant identified	not applicable						
18008440	male	5	retinal dystrophy; unilateral retinal detachment	no	177	no clearly causative variant identified	not applicable						
18012137	male	3	retinal dystrophy; unilateral congenital cataract	no	177	no clearly causative variant identified	not applicable						
17001549	female	3	macular dystrophy	no	177	no clearly causative variant identified	not applicable						
13016423	male	1	retinal dystrophy; global developmental delay; truncal hypotonia	no	genome sequencing	POMGNT1 c.1342G>C p.(Gly448Arg) het; POMGNT1 c.1769G>A p.(Trp590Ter) het	class 4 (likely pathogenic); class 5 (pathogenic)					family interested in pre-implantation genetic diagnosis	yes [PMID:28341476]
12009678	female	4	retinal dystrophy; developmental delay; cerebellar atrophy	no	177	no clearly causative variant identified	not applicable						yes [PMID:28341476]
14012857	female	5	retinal dystrophy; polydactyly; obesity; vaginal & anal atresia	no	genome sequencing	no clearly causative variant identified	not applicable						yes [PMID:28341476]
17030562	female	4	retinal dystrophy; digit abnormalities; obesity	no	177	no clearly causative variant identified	not applicable						
14021804	male	2	retinoschisis	yes	177	RS1 c.592_600dup p.(Arg200_Leu201insPheIleArg) hemi	class 3 (VOUS)					surveillance for retinal detachment initiated	yes [PMID:28341476]
17029101	male	2	retinoschisis	no	177	RS1 c.305G>A p.(Arg102Gln) hemi	class 4 (likely pathogenic)						

labno	gender	age at referral for genetic testing in years	referral diagnosis	relevant family history	number of genes tested	genotype	ACMG variant classification	avoiding unneccessary tests	initiating surveillance for extraocular manifestations	reducing prognostic uncertainty	determining eligibility for treatment trials	other / comment	case previously reported by our group
17018420	male	<1	vitreoretinal dysplasia	no	177	RS1 c.35T>A p.(Leu12His) hemi	class 4 (likely pathogenic)					surveillance for retinal detachment initiated	
12000347	male	1	exudative vitreoretinopathy	no	105	FZD4 c.313A>G p.(Met105Val) het	class 4 (likely pathogenic)						
17011544	female	4	exudative vitreoretinopathy	no	177	no clearly causative variant identified	not applicable						
15023722	female	1	vitreoretinal dysplasia; microcephaly; developmental delay	no	177	KIF11 c.478_479del p.(Leu160ValfsTer5) het; NPHP4 c.271T>C p.(Phe91Leu) het	class 5 (pathogenic); class 3 (VOUS)						yes [PMID:28341476]
16004891	female	2	vitreoretinal dysplasia; porencephaly; developmental delay	no	177	no clearly causative variant identified	not applicable						
14009949	female	4	vitreoretinopathy; microcephaly	no	177	no clearly causative variant identified	not applicable						

^ ACMG corresponds to the American College of Medical Genetics and Genomics (the variant classification shown is according to PMID: 25741868);
VOUS corresponds to variant of unknown significance

Supplementary Table 2: Genes and transcripts included in panels that were used to test children with bilateral cataracts, ectopia lentis or anterior segment dysgenesis (114 gene panel; samples tested between September 2011 and August 2016)

Gene (HGNC)	Transcript	Gene (HGNC)	Transcript	Gene (HGNC)	Transcript	Gene (HGNC)	Transcript
ADAMTSL4	NM_019032.4	*ERCC8*	NM_000082.3	*NDP*	NM_000266.3	*SC5DL*	NM_001024956.2
AGK	NM_018238.3	*EYA1*	NM_172058.2; NM_172060.2	*NF2*	NM_000268.3; NM_016418.5; NM_181832.2	*SEC23A*	NM_006364.2
AGPS	NM_003659.3	*FAM126A*	NM_032581.3	*NHS*	NM_198270.2; NM_001136024.2	*SIL1*	NM_001037633.1
AKR1E2	NM_001040177.1	*FBN1*	NM_000138.4	*OCRL*	NM_000276.3	*SIX5*	NM_175875.4
ALDH18A1	NM_002860.3	*FKRP*	NM_001039885.2	*OPA3*	NM_001017989.2	*SIX6*	NM_007374.2
B3GALTL	NM_194318.3	*FKTN*	NM_006731.2	*PAX6*	NM_001604.4	*SLC16A12*	NM_213606.3
BCOR	NM_017745.5; NM_001123385.1; NM_001123384.1	*FOXC1*	NM_001453.2	*PEX1*	NM_000466.2	*SLC2A1*	NM_006516.2
BFSP1	NM_001195.3	*FOXD3*	NM_012183.2	*PEX12*	NM_000286.2	*SLC33A1*	NM_004733.3
BFSP2	NM_003571.2	*FOXE3*	NM_012186.2	*PEX13*	NM_002618.3	*SOLH*	NM_005632.2
CBS (ABHD5)	NM_000071.2	*FTL*	NM_000146.3	*PEX16*	NM_057174.2; NM_004813.2	*SOX2*	NM_003106.3
CHMP4B	NM_176812.4	*FYCO1*	NM_024513.2	*PEX2*	NM_001172086.1	*SRD5A3*	NM_024592.4
COL18A1	NM_130445.2	*FZD4*	NM_012193.3	*PEX26*	NM_017929.5	*SREBF2*	NM_004599.2
COL2A1	NM_001844.4	*GALK1*	NM_000154.1	*PEX3*	NM_003630.2	*TDRD7*	NM_014290.2
COL4A1	NM_001845.4	*GALT*	NM_000155.2	*PEX6*	NM_000287.3	*TFAP2A*	NM_003220.2; NM_001042425.1; NM_001032280.2
COL11A1	NM_001854.3	*GCNT2*	NM_145649.4; NM_145655.3; NM_001491.2	*PEX7*	NM_000288.3	*TMEM70*	NM_017866.5
CRYAA	NM_000394.2	*GJA1*	NM_000165.3	*PEX5L*	NM_016559.2	*TMEM114*	NM_001146336.1
CRYAB	NM_001885.1	*GJA3*	NM_021954.3	*PEX10*	NM_153818.1	*VIM*	NM_003380.3
CRYBA1	NM_005208.4	*GJA8*	NM_005267.4	*PEX11B*	NM_003846.2	*VSX2 (CHX10)*	NM_182894.2
CRYBA4	NM_001886.2	*GNPAT*	NM_014236.3	*PEX14*	NM_004565.2		
CRYBB1	NM_001887.3	*HMX1*	NM_018942.2	*PEX19*	NM_002857.3		
CRYBB2	NM_000496.2	*HSF4*	NM_001040667.2	*PITX2*	NM_000325.5; NM_153426.1		
CRYBB3	NM_004076.3	*JAM3*	NM_032801.3	*PITX3*	NM_005029.3		
CRYGC	NM_020989.3	*LARGE*	NM_004737.4	*POMT1*	NM_007171.3		
CRYGD	NM_006891.3	*LMX1B*	NM_001174146.1	*POMT2*	NM_013382.5		
CRYGS	NM_017541.2	*LRP5*	NM_002335.2	*PVRL3*	NM_015480.1		
CYP27A1	NM_000784.3	*LTBP2*	NM_000428.2	*PXDN*	NM_012293.1		
CYP51A1	NM_000786.3	*MAF*	NM_005360.4	*RAB18*	NM_021252.3		
DHCR7	NM_001360.2	*MAN2B1*	NM_002372.2	*RAB3GAP1*	NM_001172435.1		
EPHA2	NM_004431.3	*MFSD6L*	NM_152599.3	*RAB3GAP2*	NM_012414.3		
ERCC2	NM_000400.3	*MIP*	NM_012064.3	*RECQL2 (WRN)*	NM_000553.4		
ERCC3	NM_000122.1	*MIR184*	NR_029705.1	*RECQL4*	NM_004260.3		

Supplementary Table 3: Genes and transcripts included in panels that were used to test children with bilateral cataracts, ectopia lentis or anterior segment dysgenesis (144 gene panel; samples tested between September 2016 and August 2018)

Gene (HGNC)	Transcript	Gene (HGNC)	Transcript	Gene (HGNC)	Transcript	Gene (HGNC)	Transcript
ABCA3	NM_001089.2	***CYP27A1***	NM_000784.3	***LEPREL1 (P3H2)***	NM_018192.3	***PVRL3***	NM_015480.1
ABHD12	NM_001042472.2; NM_015600.4	***CYP51A1***	NM_000786.3	***LIM2***	NM_030657.3	***PXDN***	NM_012293.1
ABHD5 (CDS)	NM_016006.4	***DHCR7***	NM_001360.2	***LONP1***	NM_004793.3	***RAB18***	NM_021252.3
ADAMTS10	NM_030957.2	***DNAJB1***	NM_006145.2; NM_001313964.1; NM_001300914.1	***LRP2***	NM_004525.2	***RAB3GAP1***	NM_001172435.1
ADAMTS17	NM_139057.2	***EBP***	NM_006579.2	***LSS***	NM_002340.5	***RAB3GAP2***	NM_012414.3
ADAMTSL4	NM_019032.4	***EIF2B2***	NM_014239.3	***LTBP2***	NM_000428.2	***RECQL2 (WRN)***	NM_000553.4
AGK	NM_018238.3	***EPG5***	NM_020964.2	***MAF***	NM_005360.4	***RECQL4***	NM_004260.3
AGPS	NM_003659.3	***EPHA2***	NM_004431.3	***MAN2A1***	NM_002372.2	***RGS6***	NM_001204416; NM_001204417.1
AKR1E2	NM_001040177.1	***ERCC2***	NM_000400.3	***MAN2B1***	NM_002372.2	***RNLS***	NM_001031709.2; NM_018363.3
ALDH18A1	NM_002860.3	***ERCC3***	NM_000122.1	***MED25***	NM_030973.3	***SC5DL***	NM_001024956.2
ATOH7	NM_145178.3	***ERCC6***	NM_000124.2	***MFSD6L***	NM_152599.3	***SEC23A***	NM_006364.2
BCOR	NM_017745.5; NM_001123385.1; NM_001123384.1	***ERCC8***	NM_000082.3	***MIP***	NM_012064.3	***SIL1***	NM_001037633.1
BFSP1	NM_001195.3	***EYA1***	NM_172058.2; NM_172060.2	***MIR184***	NR_029705.1	***SIPA1L3***	NM_015073.2
BFSP2	NM_003571.2	***FAM126A***	NM_032581.3	***MVK***	NM_000431.2	***SIX5***	NM_175875.4
CAV1	NM_001753.4	***FAR1***	NM_032228.5	***MYH9***	NM_002473.4	***SLC16A12***	NM_213606.3
CBS	NM_00071.2	***FBN1***	NM_000138.4	***NDP***	NM_000266.3	***SLC2A1***	NM_006516.2
CHMBP4B	NM_176812.4	***FKRP***	NM_001039885.2	***NF2***	NM_000268.3; NM_016418.5; NM_181832.2	***SLC33A1***	NM_004733.3
CHRDL1	NM_001143981	***FKTN***	NM_006731.2	***NHS***	NM_198270.2; NM_001136024.2	***SOLH***	NM_005632.2
CHX10 (VSX2)	NM_182894.2	***FOXD3***	NM_012183.2	***OAT***	NM_000274.3	***SRD5A3***	NM_024592.4
CLPB	NM_030813.4; NM_001258394.2	***FOXE3***	NM_012186.2	***OCRL***	NM_000276.3	***SREBF2***	NM_004599.2
COL11A1	NM_001854.3	***FTL promoter***	NM_000146.3	***OPA3***	NM_001017989.2; NM_025136.3	***TBC1D20***	NM_144628.3
COL18A1	NM_130445.2	***FYCO1***	NM_024513.2	***OTX2***	NM_021728.3	***TDRD7***	NM_014290.2
COL2A1	NM_001844.4	***GALK1***	NM_000154.1	***PAX6***	NM_001604.4	***TFAP2A***	NM_003220.2; NM_001042425.1; NM_001032280.2
COL4A1	NM_001845.4	***GALT***	NM_000155.2	***PEX1***	NM_000466.2	***TMEM114***	NM_001146336
CRYAA	NM_000394.2	***GBA2***	NM_020944.2	***PEX12***	NM_000286.2	***TMEM70***	NM_017866.5
CRYAB	NM_001885.1	***GCNT2***	NM_145649.4; NM_145655.3; NM_001491.2	***PEX13***	NM_002618.3	***TRAPPC11***	NM_021942.5
**CRYBA1**	NM_005208.4	***GFER***	NM_005262.2	***PEX16***	NM_057174.2; NM_004813.2	***TRPM3***	NM_001007471.2; NM_001007471.2; NM_020952.4; NM_024971
CRYBA2	NM_005209.1	***GJA1***	NM_000165.3	***PEX2***	NM_001172086.1	***UNC45B***	NM_173167.3
CRYBA4	NM_001886.2	***GJA3***	NM_021954.3	***PEX26***	NM_017929.5	***VIM***	NM_003380.3
CRYBB1	NM_001887.3	***GJA8***	NM_005267.4	***PEX3***	NM_003630.2	***VSX2***	NM_182894.2
CRYBB2	NM_000496.2	***GNPAT***	NM_014236.3	***PEX6***	NM_000287.3	***WFS1***	NM_006005.3
CRYBB3	NM_004076.3	***GTF2H5***	NM_207118.2	***PEX7***	NM_000288.3	***XYLT2***	NM_022167.3
CRYGB	NM_005210.3	***HMX1***	NM_018942.2	***PITX2***	NM_000325.5, NM_153426.1		
CRYGC	NM_020989.3	***HSF4***	NM_001040667.2	***PITX3***	NM_005029.3		
CRYGD	NM_006891.3	***JAM3***	NM_032801.3	***POMGNT1***	NM_017739.3; NM_001243766.1		
CRYGS	NM_017541.2	***LAMB2***	NM_002292.3	***POMT1***	NM_007171.3		
CTDP1	NM_004715.4	***LARGE***	NM_004737.4	***POMT2***	NM_013382.5		

Supplementary Table 4: Genes and transcripts included in panels that were used to test children with corneal dystrophies or anterior segment dysgenesis, including pediatric glaucoma (samples tested between September 2015 and August 2018)

**Corneal abnormalities panel** (32 genes)		**Anterior segment dysgenesis & glaucoma panel** (45 genes)			
**Gene (HGNC)**	**Transcript**	**Gene (HGNC)**	**Transcript**	**Gene (HGNC)**	**Transcript**
ABCA3	NM_001089.2	***ADAMTS10***	NM_030957.2	***PEX2***	NM_001172086.1
ADAMTS18	NM_199355.2	***ADAMTS17***	NM_139057.2	***PEX26***	NM_017929.5
ADAMTSL4	NM_019032.4	***ATOH7***	NM_145178.3	***PEX3***	NM_003630.2
ALDH18A1	NM_002860.3	***B3GALTL***	NM_194318.3	***PEX6***	NM_000287.3
B3GALTL (B3GLCT)	NM_194318.3	***(B3GLCT)***		***PEX7***	NM_000288.3
CHRDL1	NM_001143981	***COL4A1***	NM_001845.4	***PIK3R1***	NM_181523.2; NM_181504.3;
CHST6	NM_021615.4	***CYP1B1***	NM_000104.3		NM_181524.1
COL8A2	NM_005202.3	***DDX58***	NM_014314.3	***PITX2***	NM_000325.5; NM_153426.1
GJA1	NM_005202.3	***DHCR7***	NM_001360.2	***PITX3***	NM_005029.3
GLS1	NM_014905.4	***DNAJB1***	NM_006145.2; NM_001313964.1;	***POMGNT1***	NM_017739.3; NM_001243766.1
HMX1	NM_018942.2		NM_001300914.1	***POMT1***	NM_007171.3
KERA	NM_007035.3	***FBN1***	NM_000138.4	***POMT2***	NM_013382.5
KRT12	NM_000223.3	***FKRP***	NM_001039885.2	***PXDN***	NM_012293.1
KRT3	NM_057088.2	***FKTN***	NM_006731.2	***SBF2***	NM_030962.3
LAMB2	NM_002292.3	***FOXC1***	NM_001453.2	***SLC38A8***	NM_001080442.2
MED25	NM_030973.3	***FOXD3***	NM_012183.2		
MIR184	NR_029705.1	***FOXE3***	NM_012186.2		
OVOL2 promoter	NM_021220.3	***GJA1***	NM_000165.3		
PIK3R1	NM_181523.2; NM_181504.3;	***GPR180***	NM_180989.5		
	NM_181524.1	***HMX1***	NM_018942.2		
PIP5K3 (PIKFYVE)	NM_015040.3;NM_001178000.1	***JAG1***	NM_000214.2		
PRDM5	NM_018699.3	***LAMB2***	NM_002292.3		
PXDN	NM_012293.1	***LMX1B***	NM_001174146.1		
RAB18	NM_021252.3	***LTBP2***	NM_000428.2		
RAB3GAP1	NM_001172435.1	***MAF***	NM_005360.4		
RAB3GAP2	NM_012414.3	***MIR184***	NR_029705.1		
SLC16A12	NM_213606.3	***MYOC (TIGR)***	NM_000261.1		
SLC4A11	NM_032034; NM_001174089.1;	***OPTN***	NM_001008211.1		
	NM_001174090.1	***PAX6***	NM_001604.4		
TACSTD2	NM_002353.2	***PEX1***	NM_000466.2		
TGFBI	NM_000358.2	***PEX12***	NM_000286.2		
UBIAD1	NM_013319.2	***PEX13***	NM_002618.3		
ZEB1	NM_030751.5;NM_001174094.1	***PEX16***	NM_057174.2; NM_004813.2		
ZNF469	NM_001127464.2	***PEX2***	NM_001172086.1		

Supplementary Table 5: Genes and transcripts included in panels that were used to test children with suspected albinism (samples tested between January 2016 and August 2018)

**Optic nerve disorders panel** (40 genes; including foveal hypolasia & ocular/oculocutaneous albinism panels)		**Foveal hypoplasia & nystagmus panel** (26 genes; including ocular/oculocutaneous albinism panel)		**Ocular/oculocutaneous albinism panel** (18 genes)	
**Gene (HGNC)**	Transcript	**Gene (HGNC)**	Transcript	**Gene (HGNC)**	Transcript
ACO2	NM_001098.2	***ACO2***	NM_001098.2	***AP3B1***	NM_003664.4
AP3B1	NM_003664.4	***AP3B1***	NM_003664.4	***BLOC1S3***	NM_212550.4
ATOH7	NM_145178.3	***ATOH7***	NM_145178.3	***C10orf11***	NM_032024.3; NM_001305581.1
BLOC1S3	NM_212550.4	***BLOC1S3***	NM_212550.4	***CACNA1F***	NM_005183.3; NM_001256790.2
C10orf11	NM_032024.3; NM_001305581.1	***C10orf11***	NM_032024.3; NM_001305581.1	***DTNBP1***	NM_032122.4; NM_183040.2
C12orf65	NM_152269.4	***CACNA1F***	NM_005183.3; NM_001256790.2	***GPR143***	NM_000273.2
CACNA1F	NM_005183.3; NM_001256790.2	***DTNBP1***	NM_032122.4; NM_183040.2	***HPS3***	NM_032383.4
CISD2	NM_001008388.4	***FRMD7***	NM_194277.2	***HPS4***	NM_022081.5; NM_152841.2
DHCR7	NM_001360.2	***GPR143***	NM_000273.2	***HPS5***	NM_181507.1
DTNBP1	NM_032122.4; NM_183040.2	***HMX1***	NM_018942.2	***HPS6***	NM_024747.5
FRMD7	NM_194277.2	***HPS3***	NM_032383.4	***LYST***	NM_000081.3
GJA1	NM_000165.3	***HPS4***	NM_022081.5; NM_152841.2	***OCA2***	NM_000275.2
GPR143	NM_000273.2	***HPS5***	NM_181507.1	***PLDN (BLOC1S6)***	NM_001311255.1; NM_012388.3
HMX1	NM_018942.2	***HPS6***	NM_024747.5	***SLC24A5***	NM_205850.2
HPS3	NM_032383.4	***LYST***	NM_000081.3	***SLC45A2***	NM_016180.4; NM_001012509.3
HPS4	NM_022081.5; NM_152841.2	***OCA2***	NM_000275.2	***SLC4A11***	NM_032034; NM_001174089.1;
HPS5	NM_181507.1	***PAX6***	NM_001604.4		NM_001174090.1
HPS6	NM_024747.5	***PLDN (BLOC1S6)***	NM_001311255.1; NM_012388.3	***TYR***	NM_000372.4
LARGE	NM_004737.4	***RTN4IP1***	NM_032730.4	***TYRP1***	NM_000550.2
LYST	NM_000081.3	***SIX6***	NM_007374.2		
OCA2	NM_000275.2	***SLC24A5***	NM_205850.2		
OPA1	NM_015560.2; NM_130837.2	***SLC38A8***	NM_001080442.1		
OPA3	NM_025136.3; NM_001017989.2	***SLC45A2***	NM_016180.4; NM_001012509.3		
PAX6	NM_001604.4	***SLC4A11***	NM_032034; NM_001174089.1;		
PLDN (BLOC1S6)	NM_001311255.1; NM_012388.3		NM_001174090.1		
RAB18	NM_021252.3	***TYR***	NM_000372.4		
RAB3GAP1	NM_001172435.1	***TYRP1***	NM_000550.2		
RAB3GAP2	NM_012414.3				
RTN4IP1	NM_032730.4				
SIX6	NM_007374.2				
SLC24A5	NM_205850.2				
SLC38A8	NM_001080442.1				
SLC45A2	NM_016180.4; NM_001012509.3				
SLC4A11	NM_032034; NM_001174089.1;				
	NM_001174090.1				
SOX2	NM_003106.3				
TBC1D20	NM_144628.3				
TMEM126A	NM_032273.3				
TYR	NM_000372.4				
TYRP1	NM_000550.2				
WFS1	NM_006005.3				

We were unable to gain acceptable coverage of HPS1 to include it on these clinical diagnostic panels. HPS10 was also not included.

Supplementary Table 6: Genes and transcripts included in panels that were used to test children with inherited retinal disorders (105 gene panel; samples tested between September 2011 and June 2014)

Gene (HGNC)	Transcript	Gene (HGNC)	Transcript	Gene (HGNC)	Transcript	Gene (HGNC)	Transcript
ABCA4	NM_000350.2	***CRB1***	NM_201253.2	***NR2E3***	NM_014249.2	***RIMS1***	NM_001168410.1
ADAM9	NM_003816.2	***CRX***	NM_000554.4	***NRL***	NM_006177.3	***RLBP1***	NM_000326.4
AIPL1	NM_014336.3	***DFNB31***	NM_015404.3	***OTX2***	NM_021728.2	***ROM1***	NM_000327.3
ARL6	NM_032146.3	***DHDDS***	NM_024887.2	***PCDH15***	NM_001142763.1	***RP1***	NM_006269.1
BBS1	NM_024649.4	***EFEMP1***	NM_001039348.2	***PCDH15***	NM_001142769.1	***RP1L1*****	NM_178857.5
BBS10	NM_024685.3	***ELOVL4***	NM_022726.3	***PCDH15***	NM_001142770.1	***RP2***	NM_006915.2
BBS12	NM_001178007.1	***EYS***	NM_001142800.1	***PCDH15***	NM_001142771.1	***RP9***	NM_203288.1
BBS2	NM_031885.3	***FAM161A***	NM_001201543.1	***PDE6A***	NM_000440.2	***RPE65***	NM_000329.2
BBS4	NM_033028.3	***FSCN2***	NM_001077182.2	***PDE6B***	NM_000283.3	***RPGR****	NM_001034853.1
BBS5	NM_152384.2	***FZD4***	NM_012193.3	***PDE6C***	NM_006204.3	***RPGRIP1***	NM_020366.3
BBS7	NM_176824.2	***GNAT2***	NM_005272.3	***PDE6G***	NM_002602.3	***RS1***	NM_000330.3
BBS7	NM_018190.3	***GPR98***	NM_032119.3	***PITPNM3***	NM_031220.3	***SAG***	NM_000541.4
BBS9	NM_198428.2	***GUCA1A***	NM_000409.3	***PRCD***	NM_001077620.2	***SEMA4A***	NM_022367.3
BEST1	NM_004183.3	***GUCA1B***	NM_002098.5	***PROM1***	NM_006017.2	***SNRNP200***	NM_014014.4
C1QTNF5	NM_015645.3	***GUCY2D***	NM_000180.3	***PRPF3***	NM_004698.2	***SPATA7***	NM_018418.4
C2orf71	NM_001029883.1	***IDH3B***	NM_006899.2	***PRPF31***	NM_015629.3	***TEAD1***	NM_021961.5
CA4	NM_000717.3	***IDH3B***	NM_174855.1	***PRPF6***	NM_012469.3	***TIMP3***	NM_000362.4
CACNA2D4	NM_172364.4	***IMPDH1***	NM_000883.3	***PRPF8***	NM_006445.3	***TOPORS***	NM_005802.4
CDH23	NM_022124.5	***IMPG2***	NM_016247.3	***PRPH2***	NM_000322.4	***TRIM32***	NM_012210.3
CDHR1	NM_001171971.1	***KCNV2***	NM_133497.3	***RAX2***	NM_032753.3	***TTC8***	NM_144596.2
CDHR1	NM_033100.2	***KLHL7***	NM_001031710.2	***RBP3***	NM_002900.2	***TULP1***	NM_003322.3
CEP290***	NM_025114.3	***LCA5***	NM_181714.3	***RD3***	NM_183059.2	***UNC119***	NM_005148.3
CERKL	NM_001030311.2	***LRAT***	NM_004744.3	***RDH12***	NM_152443.2	**UNC119**	NM_054035.2
CHM	NM_000390.2	***LRP5***	NM_002335.2	***RDH5***	NM_001199771.1	***USH1C***	NM_005709.3
CLRN1	NM_052995.2	***MERTK***	NM_006343.2	***RGR***	NM_002921.3	***USH1C***	NM_153676.3
CLRN1	NM_001195794.1	***MKKS***	NM_018848.2	***RGS9***	NM_003835.3	***USH1G***	NM_173477.2
CNGA1	NM_001142564.1	***MKS1***	NM_017777.3	***RGS9***	NM_001165933.1	***USH2A***	NM_206933.2
CNGA3	NM_001298.2	***MKS1***	NM_001165927.1	***RHO***	NM_000539.3	***ZNF513***	NM_144631.5
CNGB1	NM_001297.4	***MYO7A***	NM_000260.3	***RIMS1***	NM_014989.4		
CNGB3	NM_019098.4	***NDP***	NM_000266.3	***RIMS1***	NM_001168407.1		

* For RPGR it was not possible to obtain high quality read for exon ORF15;
** Analysis of the coding region between c.4000 and c.7000 of the RP1L1 gene was not included;
***Testing of the common intron 26 mutation c.2991+1655A>G in CEP290 was included in this analysis.

Supplementary Table 7: Genes and transcripts included in panels that were used to test children with inherited retinal disorders (177 gene panel; samples tested between July 2014 and August 2018)

Gene (HGNC)	Transcript	Gene (HGNC)	Transcript	Gene (HGNC)	Transcript	Gene (HGNC)	Transcript
ABCA4*	NM_000350	***CRX***	NM_000554	***MFRP***	NM_031433	***RGR***	NM_002921
ABHD12	NM_001042472	***CSPP1***	NM_024790	***MKKS***	NM_018848	***RGS9***	NM_003835
ACBD5	NM_145698	***CYP4V2***	NM_207352	***MKS1***	NM_017777	***RGS9***	NM_001165933
ADAM9	NM_003816	***DFNB31***	NM_015404	***MKS1***	NM_001165927	***RHO***	NM_000539
ADAMTS18	NM_199355	***DHDDS***	NM_024887	***MVK***	NM_000431	***RIMS1***	NM_014989
AHI1	NM_001134832	***DTHD1***	NM_001136536	***MYO7A***	NM_000260	***RIMS1***	NM_001168407
AHI1	NM_017651	***DTHD1***	NM_001170700	***NDP***	NM_000266	***RIMS1***	NM_001168410
AIPL1	NM_014336	***EFEMP1***	NM_001039348	***NEK2***	NM_002497	***RLBP1***	NM_000326
ARL2BP	NM_012106	***ELOVL4***	NM_022726	***NMNAT1***	NM_0022787	***ROM1***	NM_000327
ARL6	NM_032146	***EMC1***	NM_015047	***NPHP1***	NM_000272	***RP1***	NM_006269
BBIP1	NM_001195306	***EYS***	NM_001142800	***NPHP3***	NM_153240	***RP1L1*****	NM_178857
BBS1	NM_024649	***FAM161A***	NM_001201543	***NPHP4***	NM_015102	***RP2***	NM_006915
BBS10	NM_024685	***FLVCR1***	NM_014053	***NR2E3***	NM_014249	***RP9***	NM_203288
BBS12	NM_001178007	***FSCN2***	NM_001077182	***NRL***	NM_006177	***RPE65***	NM_000329
BBS2	NM_031885	***FZD4***	NM_012193	***NYX***	NM_022567	***RPGR******	NM_001034853
BBS4	NM_033028	***GNAT1***	NM_000172	***OAT***	NM_000274	***RPGRIP1***	NM_020366
BBS5	NM_152384	***GNAT2***	NM_005272	***OFD1****	NM_003611	***RPGRIP1L***	NM_015272
BBS7	NM_176824	***GNPTG***	NM_032520	***OTX2***	NM_021728	***RS1***	NM_000330
BBS9	NM_198428	***GPR125***	NM_145290	***PANK2***	NM_153638	***SAG***	NM_000541
BEST1	NM_004183	***GPR179***	NM_001004334	***PCDH15***	NM_001142763	***SDCCAG8***	NM_006642
C1QTNF5	NM_015645	***GPR98***	NM_032119	***PCDH15***	NM_001142769	***SEMA4A***	NM_022367
C2orf71	NM_001029883	***GRK1***	NM_002929	***PCDH15***	NM_001142770	***SLC24A1***	NM_004727
C2ORF86	NM_015910	***GRM6***	NM_000843	***PCDH15***	NM_001142771	***SLC24A1***	NM_001254740
C8ORF37	NM_177965	***GUCA1A***	NM_000409	***PCYT1A***	NM_005017	***SNRNP200***	NM_014014
C21orf2	NM_004928	***GUCA1B***	NM_002098	***PDE6A***	NM_000440	***SPATA7***	NM_018418
CA4	NM_000717	***GUCY2D***	NM_000180	***PDE6B***	NM_000283	***TEAD1***	NM_021961
CABP4	NM_145200	***HARS***	NM_002109	***PDE6C***	NM_006204	***TIMP3***	NM_000362
CACNA1F	NM_005183	***HMX1***	NM_018942	***PDE6G***	NM_002602	***TMEM237***	NM_001044385
CACNA2D4	NM_172364	***IDH3B***	NM_006899	***PEX1***	NM_000466	***TOPORS***	NM_005802
CAPN5	NM_004055	***IDH3B***	NM_174855	***PEX2 (PXMP3)***	NM_000318	***TRIM32***	NM_012210
CC2D2A	NM_001080522	***IFT140***	NM_014714	***PEX7***	NM_000288	***TRPM1***	NM_002420
CDH3	NM_001793	***IMPDH1***	NM_000883	***PHYH***	NM_006214	***TSPAN12***	NM_012338
CDH23	NM_022124	***IMPG1***	NM_001563	***PITPNM3***	NM_031220	***TTC8***	NM_144596
CDHR1	NM_001171971	***IMPG2***	NM_016247	***PLA2G5***	NM_000929	***TUB***	NM_177972
CDHR1	NM_033100	***INPP5E***	NM_019892	***PRCD***	NM_001077620	***TULP1***	NM_003322
CEP164	NM_014956	***INVS***	NM_014425	***PROM1***	NM_006017	***UNC119***	NM_005148
CEP290*	NM_025114	***IQCB1***	NM_001023570	***PRPF3***	NM_004698	***UNC119***	NM_054035
CERKL	NM_001030311	***ITM2B***	NM_021999	***PRPF31***	NM_015629	***USH1C***	NM_005709
CHM	NM_000390	***KCNJ13***	NM_002242	***PRPF4***	NM_004697	***USH1C***	NM_153676
CIB2	NM_006383	***KCNV2***	NM_133497	***PRPF6***	NM_012469	***USH1G***	NM_173477
CLN3	NM_000086	***KIAA1549***	NM_001164665	***PRPF8***	NM_006445	***USH2A****	NM_206933
CLRN1	NM_052995	***KIAA1549***	NM_020910	***PRPH2***	NM_000322	***VCAN***	NM_004385
CLRN1	NM_001195794	***KIF11***	NM_004523	***RAB28***	NM_001017979	***VPS13B***	NM_017890
CNGA1	NM_001142564	***KLHL7***	NM_001031710	***RAX2***	NM_032753	***WDR19***	NM_025132
CNGA3	NM_001298	***LCA5***	NM_181714	***RBP3***	NM_002900	***ZNF423***	NM_015069
CNGB1	NM_001297	***LRAT***	NM_004744	***RBP4***	NM_006744	***ZNF513***	NM_144631
CNGB3	NM_019098	***LRP5***	NM_002335	***RD3***	NM_183059		
CNNM4	NM_020184	***LZTFL1***	NM_020347	***RDH12***	NM_152443		
CRB1	NM_201253	***MERTK***	NM_006343	***RDH5***	NM_001199771		

*Testing for the following intronic mutations was included in this analysis: *CEP290* c.2991+1655A>G; *USH2A* c.7595-2144A>G; *OFD1* c.935+706A>G; *ABCA4* c.5196+1056A>G, c.5196+1137G>A, c.5196+1216C>A, c.4539+2001G>A, c.4539+2028C>T, c.5461-10T>C;
Analysis of the coding region of exon 4 of the RP1L1 gene was not included; (iv)* For *RPGR* it was not possible to obtain high quality read for exon ORF15.

Supplementary Table 8: Modes of inheritance observed across 129 children with inherited eye disease and a probable molecular diagnosis.

Mode of Inheritance	Number of occurrences	Percentage of cases (%)
**Autosomal dominant**	35	27 %
no relevant family history reported in	24	18 %
**Autosomal recessive**	79	61 %
cases with a homozygous change	38	29 %
cases with presumed compound heterozygous changes	41	32 %
**X-linked**	15	12 %
no relevant family history reported in	9	7 %
